# Peptide‐Induced Division of Polymersomes for Biomimetic Compartmentalization

**DOI:** 10.1002/anie.202413089

**Published:** 2024-11-14

**Authors:** Heloísa Bremm Madalosso, Shoupeng Cao, Tsvetomir Ivanov, Marina de Souza Melchiors, Kaloian Koynov, Camila Guindani, Pedro Henrique Hermes de Araújo, Claudia Sayer, Katharina Landfester, Lucas Caire da Silva

**Affiliations:** ^1^ Department of Physical Chemistry of Polymers Max Planck Institute for Polymer Research Ackermannweg 10 55128 Mainz Germany; ^2^ Department of Chemical Engineering and Food Engineering Federal University of Santa Catarina, Campus Trindade 88040-900 Florianópolis Brazil; ^3^ Chemical Engineering Program/ COPPE Federal University of Rio de Janeiro, Cidade Universitária Rio de Janeiro 21941-972 RJ Brazil; ^4^ Department of Chemistry McGill University 801 Sherbrooke Street West Montreal QC H3A 0B8 Canada

**Keywords:** Biomimetic systems, Polymersomes, Vesicle-division, Functional peptides, Multi-compartmentalization

## Abstract

Polymersomes are synthetic vesicles that mimic the architecture of cellular compartments such as the cell membrane and organelles. These biomimetic compartments facilitate the creation of cell‐like chemical systems, including microreactors and synthetic organelles. However, the construction of hierarchical multi‐compartment systems remains challenging and typically requires the encapsulation of pre‐formed vesicles within a host compartment. Here, we report the formation of multicompartment polymersomes with a vesicle‐in‐vesicle architecture achieved through self‐division induced by short peptides incorporated into the vesicle membrane. A phenylalanine‐phenylalanine‐methionine (FFM) tripeptide was designed and encapsulated into the polymersome via microfluidics. We demonstrate that vesicle self‐division occurs due to peptide incorporation into the membrane in response to pH changes. This self‐division creates internal vesicles capable of colocalizing enzymes. The hybrid polymer‐peptide system described here provides a straightforward method for developing subcompartmentalized systems, paving the way for engineering microreactors with life‐like properties.

## Graphical abstract

Polymersomes, synthetic vesicles mimicking cellular compartments, enable the creation of cell‐like chemical systems such as microreactors and synthetic organelles. We report multicompartment polymersomes with a vesicle‐in‐vesicle architecture formed through self‐division, driven by the pH‐triggered incorporation of a phenylalanine‐phenylalanine‐methionine tripeptide into the membrane. This process produces internal vesicles that colocalize enzymes, offering a method for engineering adaptive microreactors.

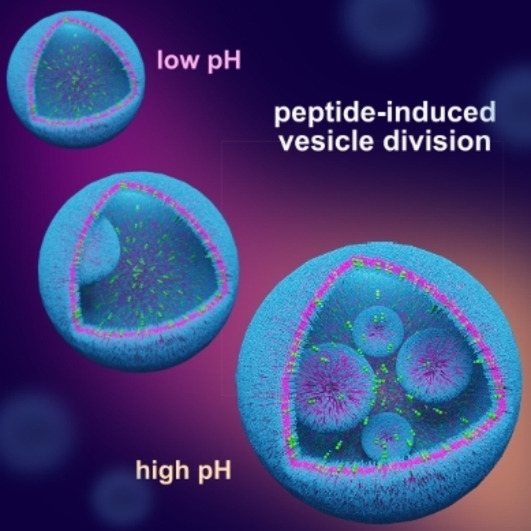

Cellular compartmentalization is a fundamental property of living cells that allows the positioning and separation of biomolecules and biochemical processes within distinct compartments.[^1^, ^2^, ^3^] This compartmentalization ensures cellular functionality and behavior, and provides the basis for the remarkable efficiency of cells as adaptive biochemical reactors.^[4]^ Inspired by this natural organization, biomimetic compartmentalized systems with life‐like properties have been developed, including microreactors, synthetic organelles, and protocells.[^5^, ^6^, ^7^] Soft biomimetic compartments, such as polymersomes,[^8^, ^9^, ^10^, ^11^, ^12^] coacervates,[^13^, ^14^, ^15^, ^16^] liposomes,[^17^, ^18^, ^19^] proteinosomes,[^20^, ^21^] colloidosomes,^[22]^ dendrimersomes,[^23^, ^24^] and combisomes^[25]^ and have attracted considerable interest in synthetic biology and materials science. These compartments offer controlled permeability, tailored surface chemistry, and efficient encapsulation of molecules and biocatalysts, making them essential tools for exploring and mimicking cellular compartmentalization in non‐biological systems.[^26^, ^27^, ^28^, ^29^, ^30^]

However, systems created using biomimetic compartments are still predominantly based on single‐compartment architectures, which are much simpler than the highly complex structures found in biological cells.[^7^, ^31^] Typically, polymeric and lipid vesicles used as compartments in cell‐like systems have a single dilute aqueous core containing the components necessary for function. To achieve enhanced functionality, such as regulation of chemical processes and dynamic response and adaptation to external stimuli, it is desirable to organize catalysts and components into multi‐compartmentalized structures.[^32^, ^33^, ^34^] To achieve this, bottom‐up approaches have been developed to create polymer and lipid vesicles with more complex internal architectures. These approaches generally rely on the encapsulation of preformed subcompartments, such as vesicles and nanoparticles, resulting in multi‐compartment structures.[^35^, ^36^, ^37^]

A less explored approach is the formation of multicompartments by vesicle division. Applying a stress to phospholipid vesicle membranes can induce vesicle splitting.[^27^, ^28^, ^38^] However, these methods typically produce independent vesicles rather than multicompartment architectures.[^39^, ^40^] A disadvantage of liposomes is their tendency to merge, which limits their ability to form stable multi‐chamber structures.[^41^, ^42^] In contrast, polymersomes made of amphiphilic copolymers are robust, making them more capable of forming stable multicompartment structures.[^43^, ^44^] Nevertheless, achieving stress‐induced division of polymersomes to form multicompartmentalized structures remains a challenge. Here, we describe the self‐division of polymersomes triggered by the stimuli‐responsive integration of short peptides into the vesicle membrane. This process induces tension in the polymeric membrane, inducing internal budding and division of polymeric vesicles in response to pH changes. Short peptides with a diphenylalanine motif are known to self‐assemble into ordered nanostructures through hydrogen bonding, π‐π stacking, and hydrophobic interactions.[^45^, ^46^]

We have recently shown that phenylalanine‐phenylalanine‐methionine (FFM), a water‐soluble peptide, condenses into metastable liquid droplets at pH > 7 before transforming into a fibrous structure^[15]^ (Figure S1). This process is controlled and initiated by deprotonation of the terminal amino group, which decreases the hydrophilicity of the peptide. In our attempts to reproduce fiber formation within polymersomes obtained from polybutadiene‐*block*‐polyethylene oxide block copolymers (PB_22_‐b‐PEO_14_), we were surprised to observe the division of the vesicles containing FFM. Vesicles were prepared using droplet microfluidics with FFM solution as the inner fluid at different concentrations (2.5, 5.0, 10 mg mL^‐1^) (Figure S3). A polymer solution in oleyl alcohol was used as the middle fluid according to a previous method.^[11]^ The expected residual amount of oleyl alcohol in the vesicle membrane is between 0.9 and 1.4% based on previously reported HPLC measurements.^[11]^ Figures 1 and S2 show a typical result where several generations of vesicles are observed. In Figure 1, the original vesicle (1) gives rise to a daughter vesicle (2), which in turn gives rise to a second generation of vesicles (3). The division was triggered at pH 7.7, indicating that a lower hydrophilicity of the peptide was required. Interestingly, peptide aggregation was only observed outside the vesicles (Figure 1c and S4), with no aggregation occurring inside. This suggested that the peptide was integrated into the polymer membrane instead of aggregating internally. To understand our observations, we investigated the distribution and interaction between FFM and the polymer membrane. This was achieved by precise encapsulation of peptides in polymersomes using a microfluidic method (Figure S3), together with confocal laser scanning microscopy and fluorescence correlation spectroscopy. First, empty vesicles were prepared and evaluated from pH 5.5 to 11.2 using confocal microscopy. In the absence of the FFM peptide, no division events were observed (Figure S5), indicating that pH alone was not sufficient to induce vesicle division. We then determined the division yield (DY) as a function of peptide concentration. DY was defined as the fraction of first‐generation vesicles that produced at least one daughter vesicle. For example, a DY of 0.5 indicates that half of the first‐generation vesicles produced at least one daughter vesicle. Counting was performed by image analysis of the samples at different conditions based on bright field microscopy. The results show that DY increased with increasing internal peptide concentration (Figure 1c). DY values were obtained at pH 7.7. A low DY of 0.08 was observed at a peptide concentration of 1 mg/mL, which reached a maximum value of 0.37 at 10 mg/mL. Changes in pH did not induce vesicle division in the absence of peptide. Higher peptide concentrations were avoided due to limitations in microfluidic vesicle production.

**Figure 1:**
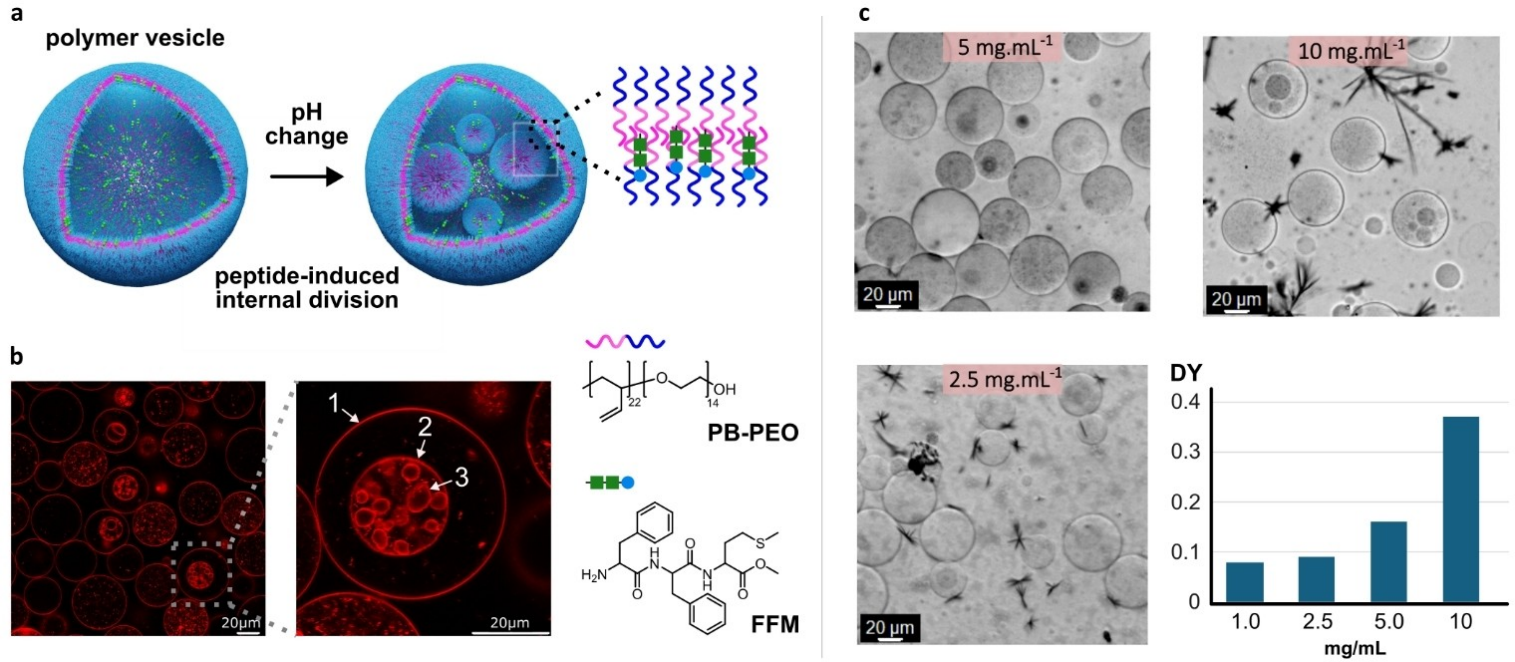
Peptide‐induced self‐division a polymersome. **(a)** Schematic illustration showing peptide insertion in the polymer membrane followed by division. **(b)** Confocal micrograph of divided vesicles at pH 7.7. Nile Red was used to stain the polymersome membrane. **(c)** Divided vesicles at different peptide (FFM) concentrations (2.5, 5 and 10 mg/mL) at pH 7.7. Division is induced even at low FFM concentrations, and the division yield (DY) increases with higher FFM concentrations at pH 7.7. Scale bar = 20 μm.

According to the differential elasticity model, differences in the areas of the inner and outer bilayer of vesicle membranes dictate vesicle morphologies.[^47^, ^48^] We hypothesize that accumulation of FFM in the inner leaflet creates an asymmetry that causes inward bulging and drives vesicle division. As shown in Movie S1, daughter vesicles are propelled by internal currents generated by changes in interfacial tension due to peptide deposition. These currents likely provide the energy needed to complete vesicle division. The effects of peptide accumulation in the membrane were studied using fluorescence correlation spectroscopy (FCS).^[49]^ This technique provided information about peptide‐induced changes in the fluidity of the polymer membranes. The data were obtained by measuring changes in the translational diffusion time (τ_D_) of a fluorescent lipophilic probe (Nile Red) embedded in the polymer membrane under two conditions: without peptide and with peptide (10 mg/mL) (Figure 2).

**Figure 2:**
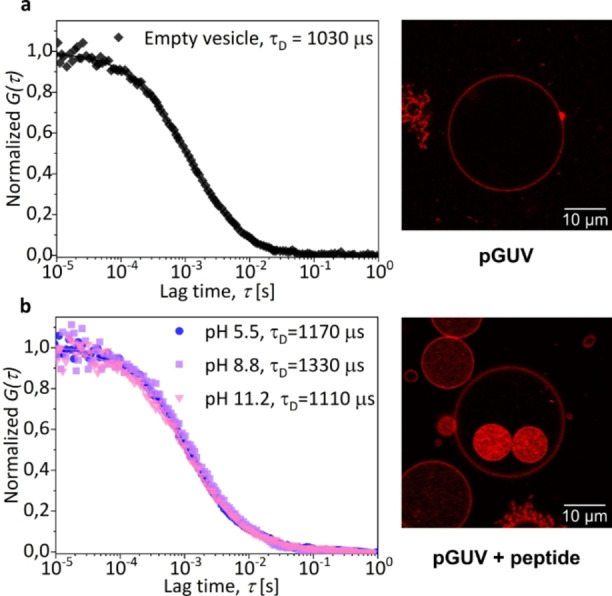
Nile Red diffusion in the membrane of polymersomes (pGUV) measured by FCS at pH 7.7. Normalized experimental autocorrelation curves (symbols) and the corresponding fits with eq. S2 **(a)** Vesicle without peptide **(b)** Vesicle containing 10 mg/mL of FFM. Scale bar = 10 μm. CLSM images were acquired at pH 7.7.

In an FCS experiment τ_D_ is the average time it takes for fluorescent probes to diffuse through the observation volume defined by the focal spot of the laser and is therefore proportional to the fluidity of the environment.[^49^, ^50^] The results show that the translational diffusion time of Nile Red in the membrane of vesicles without FFM (1030 μs) was about 1.3 times shorter than that of vesicles containing FFM at pH 8.8. This indicates that the presence of the peptide decreases the fluidity of the membrane. The increased concentration of molecules in the membrane results in an increase in the translational diffusion time of Nile Red in the presence of FFM. The translational diffusion time of Nile Red in the membrane of polymersomes reached a maximum value at pH 8.8, which is consistent with the increased hydrophobicity of the peptide. Beyond pH 5.6, the isoelectric point of FFM (Table S4), the peptide becomes increasingly neutral in solution. This reduces the solvation of FFM, causing the peptide to migrate to the membrane and localize in the hydrophobic layer. The FCS data suggests that at pH 8.8, the more hydrophobic peptide penetrates the polymeric membrane, limiting molecular mobility within the membrane. Cholesterol is known to cause a similar decrease in the fluidity of phospholipid membranes.[^51^, ^52^, ^53^] The presence of cholesterol in polymersomes also reduced the mobility of Nile Red relative to the values obtained with empty vesicles (Figure S6). However, cholesterol did not induce vesicle division. This suggests that a sudden change in the composition of the polymeric membrane, due to pH‐triggered migration and membrane insertion of FFM, is required to generate the transient tension that is eventually released through vesicle division or rupture. Since cholesterol is present during vesicle formation, there is no sudden change in membrane composition. The migration and insertion of FFM molecules increase the density of molecules in the vesicle membrane. The excess area is shed from the affected vesicles by the formation of daughter vesicles. The presence of peptides inside or outside the parent vesicles determines the direction of vesicle division. This was tested by adding the FFM peptide outside the vesicles at different pH values (Figure S13). When peptides are present outside the vesicles, division primarily occurs outward, meaning that daughter vesicles form from the surface facing the external solution rather than the inner vesicle volume, as shown in Figure 1. This effect is summarized in Figure S14. Interestingly, encapsulation of FFM in liposomes also induced internal division (Figure S7), although less efficiently. This lower performance can be explained by a lower concentration of peptides inside the more permeable liposomes due to leakage during vesicle formation.

We then investigated the effect of pH on the division yield (Figure 3). Signs of division were observed at pH 5.5 (DY = 0.05). The division yield increased significantly with increasing pH and reached a maximum at pH 8.8 (DY = 0.40, p<0.05, Figure 3). Consistently, this pH corresponds to the slowest translational diffusion of Nile Red (Figure 2). Our results suggest that vesicle division is more efficient when a higher number of FFM molecules migrate to the membrane. Beyond pH 8.8, DY is significantly reduced (2.5‐fold). The pH‐induced migration and insertion of peptides into the membrane was further studied by labeling FFM with nitrobenzoxadiazole (NBD), a small, neutral dye widely used as a tag for peptides and proteins. NBD exhibits green fluorescence in hydrophobic media (Scheme S1). Similar to FFM, FF‐NBD aggregates at pH above 7.7 (Figures S9 to S11). Both the tendency to aggregate and the size of the clusters increased with increasing pH and peptide concentration (Figure S12). In contrast with FFM, no distinct fiber‐like structures were observed outside the vesicles. This indicates that although the tendency to aggregate remains pH dependent, FF‐NBD does not form well‐defined fibers as observed with FFM (Figure 1 and S4). Figure 4 and Figure S15 show the presence of small aggregates of peptides that could not be integrated into the membrane. The distribution of FF‐NBD inside the vesicles and its translational diffusion were investigated by confocal laser microscopy and FCS (Figure 4).

**Figure 3:**
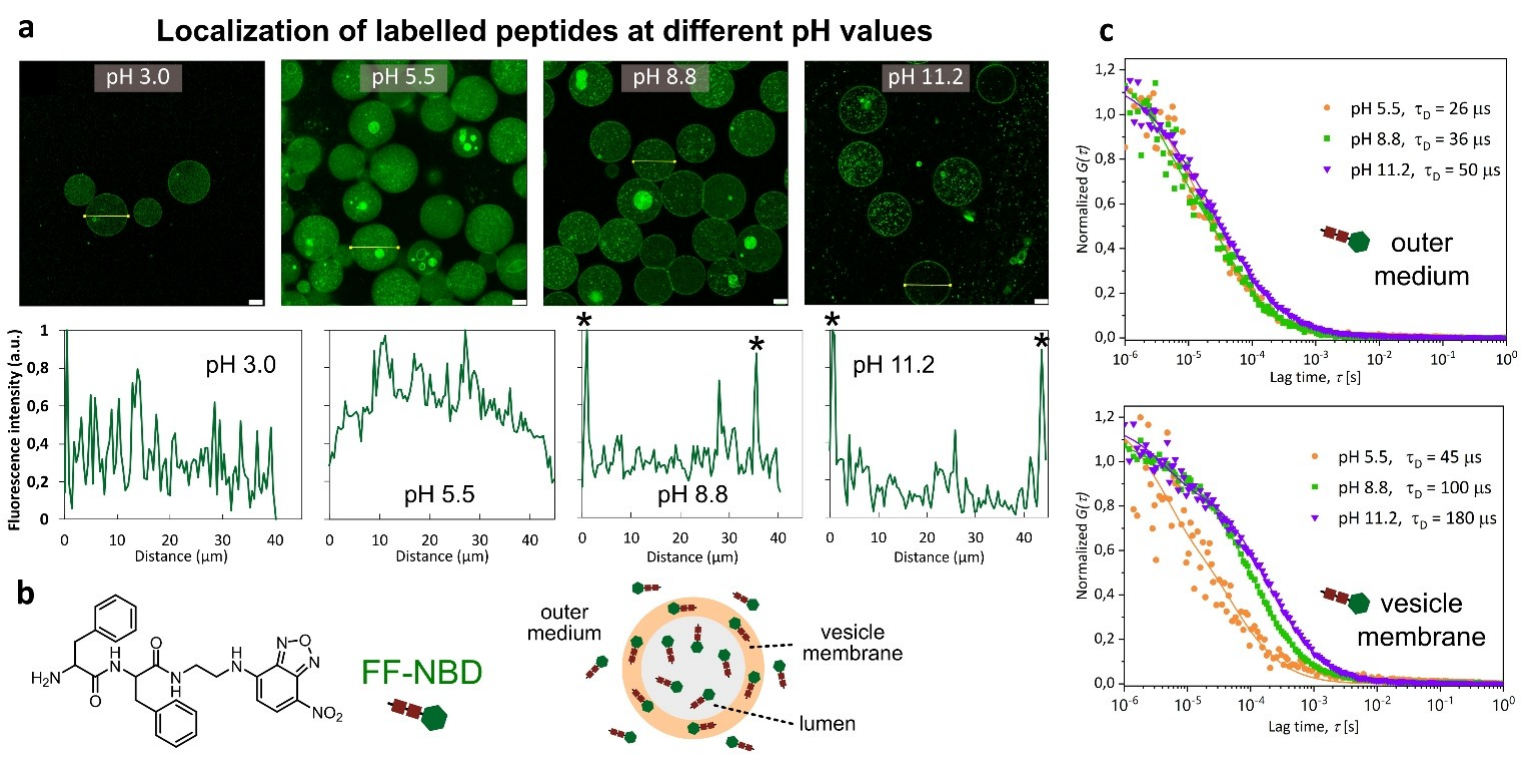
pH‐dependent distribution of peptides within polymersomes and its effect on membrane fluidity. (a) Confocal images and fluorescence line profile of polymersomes containing 10 mg/mL FFM + FF‐NBD (95 wt% FFM + 5 wt% FFM‐NBD). FF‐NBD refers to FF labeled with the fluorescent NBD moiety. The data show the distribution of the peptide at different pH values based on the fluorescence from FF‐NBD. The peaks marked with an asterisk represent membrane. Scale bar = 20 μm. (b) The structure of the FF‐NBD and a schematic representation of its distribution relative to a polymer vesicle. (c) Normalized FCS autocorrelation curves (symbols) and the corresponding fits with eq. S1 or S2 recorded at different pH values for the fluorescently labeled peptide FF‐NBD present in the outer medium (top) and localized in the vesicle membrane (bottom).

**Figure 4:**
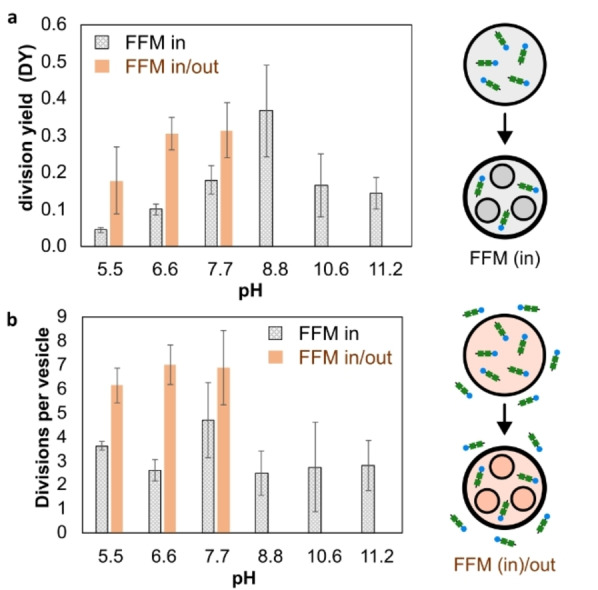
The influence of pH and presence of peptide in and outside the vesicles. (a) Yield of division (YD). (b) Number of daughter vesicles produced per mother vesicle. The error bar represents the standard deviation of the average values obtained from 3 independent measurements.

At pH 5.5, fluorescence intensity analysis shows that FFM‐NBD is not preferentially localized in the polymersome membrane. This changes at pH 8.8, where the higher emission intensity is localized in the polymersome membrane, consistent with peptide migration and membrane insertion (Figure 4a). As observed for Nile Red, the translational diffusion of the labelled peptide in the polymersome membrane was also pH dependent (Figure 4c). While the translational diffusion time of FFM‐NBD in the lumen of the vesicles remained low (26‐50 μs), a pronounced reduction in molecular mobility was observed for peptides in the membrane (45‐180 μs). It is also interesting to note that FFM‐NBD (t_D_ = 100 μs, pH 8.8) has a higher mobility in the polymersome membrane than Nile Red (t_D_ = 1330 μs, pH 8.8) even though its molecular weight is about 1.5 times greater than that of Nile Red. This result is consistent with the more hydrophobic nature of Nile Red and its low solvation compared to the peptide. While Nile Red is efficiently localized in the hydrophobic layers of the membrane, the peptide remains relatively soluble, as indicated by the residual fluorescence detected in the lumen of the vesicles. Therefore, peptide molecules may adsorb/desorb to the membrane multiple times during their passage through the FCS confocal observation volume. In such cases, FCS provides a diffusion time that is an average of the diffusion times of the bound and unbound states.^[54]^ Since FF‐NDB is more soluble than NR, the diffusion times obtained by FCS for FF‐NBD reflect the large mobility in the dilute phase and the restricted mobility in the membrane, resulting in an overall shorter average diffusion time than NR, which is mainly confined to the less mobile vesicle membrane.

A unique feature of biological cells is their ability to localize biomolecules in different subcompartments (organelles).[^7^, ^55^] Peptide‐induced self‐division of polymersomes allows the engineering of synthetic systems that mimic the multicompartmentalization of cells. To demonstrate this, labeled glucose oxidase and horseradish peroxidase enzymes (GOx‐FITC and HRP‐Cy5) and FFM were encapsulated in polymersomes using microfluidics. During the formation of new daughter vesicles, some of the enzymes originally present in the mother vesicle were detected inside the daughter vesicles, as suggested by CLSM images (Figures 5 and S16). When Amplex Red was added to the system, a cascade enzymatic reaction produced resorufin (Figure S18). Peptide‐induced division did not affect enzyme activity, highlighting the potential of our system to act as a multi‐compartment microreactor.

**Figure 5:**
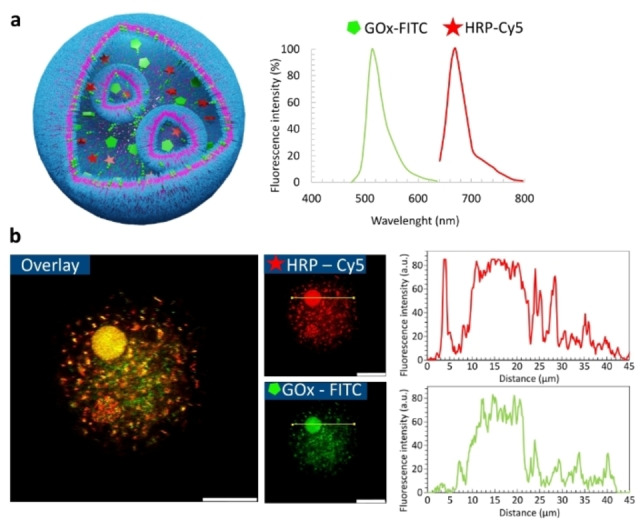
Confocal micrographs showing enzyme colocalization in internal vesicles generated by peptide‐induced self‐division. (a) Schematic representation of a multicompartment polymersome and emission spectra of tagged enzymes, glucose oxidase (GOx) and horseradish peroxidase (HRP). (b) Multicompartment polymersome showing the colocalization of HRP and Gox inside daughter vesicle. Scale bar = 20 μm.

In conclusion, our study demonstrates that pH‐triggered integration of the short peptide FFM into the membrane of a polymersome can induce internal vesicle division. This process results in the formation of multi‐compartmentalized polymersomes, with internal compartments. Such dynamic adaptability enhances the potential of polymersomes as cell‐like systems in biotechnological applications where responsiveness to external stimuli is critical. Furthermore, the ability of a short peptide to induce division in both polymersomes and liposomes suggests that vesicle division may have played a role in prebiotic processes, potentially contributing to the evolution of life and the mechanisms employed by early cells. Future developments in this area could exploit the continuous supply of polymers to support growth and division, thereby enhancing the capabilities of subcompartmentalization within these systems. This work paves the way for further exploration of polymersomes as versatile tools in biotechnology and synthetic biology, providing new insights into the design of adaptive and self‐organizing materials.

## Supporting Information

Supporting Information – Word File

Movie S1 – Polymersome division occurring at pH 7.7

Movie S2 – 3D reconstruction of multicompartment polymersome

Movie S3 – Polymersome division occurring at pH 7.7 in a chamber containing 10 mg/mL of FFM peptide inside and outside the vesicle.

## Conflict of Interests

The authors declare no conflict of interest.

## Supporting information

As a service to our authors and readers, this journal provides supporting information supplied by the authors. Such materials are peer reviewed and may be re‐organized for online delivery, but are not copy‐edited or typeset. Technical support issues arising from supporting information (other than missing files) should be addressed to the authors.

Supporting Information

Supporting Information

Supporting Information

Supporting Information
