# Supplementary material for: Peptide‐Induced Division of Polymersomes for Biomimetic Compartmentalization
Source: Angew Chem Int Ed Engl. 2024 Nov 14;63(52):e202413089. doi: 10.1002/anie.202413089 (PMC11656130; doi:10.1002/anie.202413089)
Supplement: Supplementary file 1 — Supporting Information [file ANIE-63-e202413089-s003.pdf]

Angewandte Chemie
Eine Zeitschrift der Gesellschaft Deutscher Chemiker

Supporting Information

Peptide-Induced Division of Polymersomes for Biomimetic Compartmentalization

H. Bremm Madalosso, S. Cao, T. Ivanov, M. de Souza Melchiors, K. Koynov, C. Guindani, P. H. Hermes de Araújo, C. Sayer, K. Landfester, L. Caire da Silva**

Supporting Information

Peptide-Induced Division of Polymersomes for Biomimetic Compartmentalization

Heloísa Bremm Madalosso[1,2] Shoupeng Cao[1], Tsvetomir Ivanov[1], Marina de Souza Melchiors[1], Kaloian Koynov[1], Camila Guindani[3], Pedro Henrique Hermes de Araújo[2], Claudia Sayer[2], Katharina Landfester[1]* and Lucas Caire da Silva[1,4]*

Table of Contents

1. Experimental Procedures 2
1.1 Chemicals 2
1.2 Synthesis of Phenylalanine-phenylalanine-methionine (FFM) and Phenylalanine-phenylalanine-methionine-nitrobenzodiazole (FFM-NBD) peptides 3
1.3 Vesicle production 3
The preparation of the polymersomes was carried out using a droplet microfluidic technique. The chips used to design the vesicles were prepared as described in the next sections. 4
1.3.1 Photolithography 4
1.3.2 PDMS chip production 4
1.3.3 Chip coating 4
1.3.4 Polymersome production 4
1.3.5 Liposome production 5
1.3.6 Microscopy 5
1.3.7 Fluorescence Confocal Spectroscopy (FCS) assays 6
1.3.8 Plate reader measurements 7
1.4 Determination of the isoelectric point of FFM peptide 7
1.5 Statistical analysis – Influence of osmotic pressure and pH in polymersome division 7
1.5.1 Influence of pH 7
1.5.2. Influence of osmotic pressure 8
1.6 Enzymatic cascade reaction inside the polymersomes 8
1.7 Evaluating the behavior of FF-NBD peptide in bulk 8
2 Statistical analyses of the influence of pH and osmolarity on polymersome division 8
2.1 Statistical analyses of the pH influence in the division yield (DY) 9
2.2 Statistical analyses of the effect of pH on the number of daughter vesicles produced per mother vesicle 10
2.3 Statistical analyses of the influence of osmolarity in the division yield (DY) 10
2.4 Statistical analyses of the effect of osmolarity on the number of daughter vesicles produced per mother vesicle 11
Supplementary figures 13
Captions for Video ..... **Fehler! Textmarke nicht definiert.**
References 25

1. Experimental Procedures

1.1 Chemicals

Amplex^{TM} red, oleyl alcohol (80-85% tech. grade), NaOH, Sucrose, Cholesterol, Sylgard 184 elastomer kit, Glucose Oxidase from *Aspergillus niger* (GOD, Type X-S, lyophilized powder, 100,000 – 250,000 units/g solid, G741-50KU) and peroxidase from horseradish (HRP, Type II, essentially salt-free, lyophilized powder, 150-250 units/mg solid, P8250-50KU) were supplied from Sigma Aldrich. The diblock copolymer poly(butadiene)-block-poly(ethylene oxide) (PB-PEO) was obtained from Polymer Source Inc (Canada). The lipid 1-palmitoyil-2-oleoyil-glycero-3-phosphocholine (POPC) was obtained from Avanti Polar Lipids. Syringes (1 ml) were purchased from Henke-Sass, Wolf GmbH, while 0.55 mm hypodermic needles and 30 M PTFE microbore tubing were purchased from Fisher Scientific GmbH Ultrapure water was used in all experiments.

1.2 Synthesis of Phenylalanine-phenylalanine-methionine (FFM) and Phenylalanine-phenylalanine-methionine-nitrobenzodiazole (FFM-NBD) peptides

Synthesis, purification, and characterization of FFM peptide was performed according to published procedures. [1]

NH_2-NBD was synthesized according to the literature [2]. The synthesis of NH_2-FF-NBD was carried out with adaptive procedures compared to the literature [1,3]. Brief synthetic procedures are illustrated below (Scheme S1):

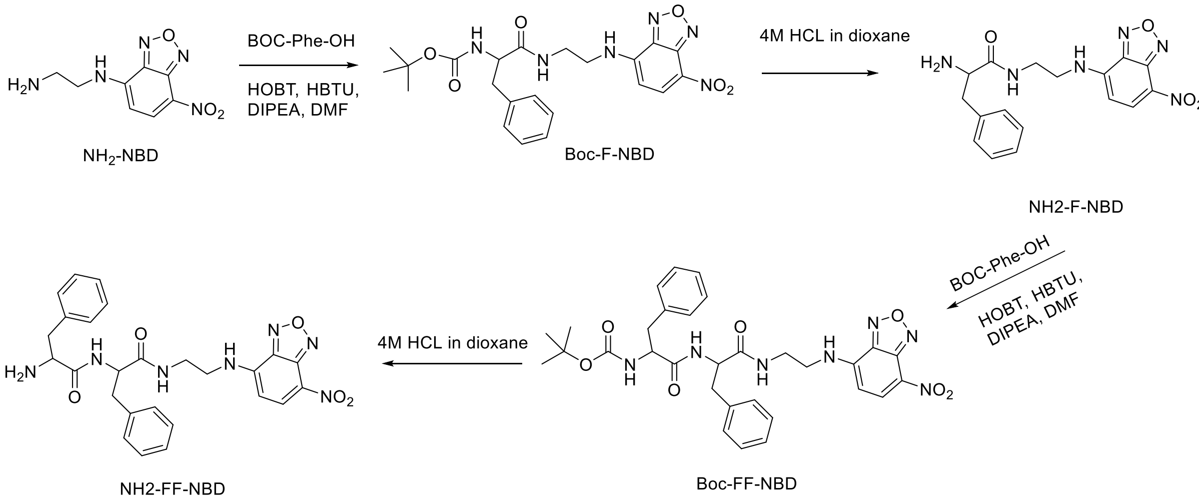

Scheme S1. Synthesis of FFM-NBD peptide

Boc-F-NBD: N-(tert-butoxycarbonyl)-L-phenylalanine (Boc-Phe-OH) (450 mg, 1.65 mmol), HBTU (624 mg, 1.65 mmol) and HOBt (225 mg, 1.65 mmol) were dissolved in DMF (5 mL) in a round bottom flask and the mixture was stirred with a magnetic stirrer. N, N-Diisopropylethylamine (DIPEA) (525 µL, 3 mmol), and NH2-NBD (400 mg, 1.5 mmol) were added at 1-minute intervals respectively and the reaction mixture was stirred for 24 h at room temperature. The reaction mixture was poured into 100 mL of water. The precipitate was collected by filtration and washed with water. The crude product was dried and further purified via column chromatography to give a 703 mg brown solid, which was characterized by NMR. ^{1}H NMR (400 MHz, DMSO-d6) δ 9.36 (s, 1H), 8.54 (d, 1H), 8.16 (t, 1H), 7.30 – 7.09 (m, 5H), 6.95 (d, 1H), 6.45 (d, 1H), 4.08 (m, 1H), 3.60 – 3.35 (m, 4H), 3.01 – 2.87 (m, 1H), 1.27 (s, 9H).

NH_2-F-NBD: The intermediate compound was dissolved in 6 mL of 4 M hydrogen chloride solution in dioxane for protection. After stirring for 3 hours, the solvent was evaporated on a rotary evaporator to give an oily residue. Diethyl ether was added to the flask and the contents were stirred gently. A white precipitate formed and was separated by centrifugation, yielding about 601 mg of a yellow product. The product was characterized by NMR. ^{1}H NMR (400 MHz, DMSO-d6) δ 9.34 (s, 1H), 8.81 (s, 1H), 8.56 (d, 1H), 8.35 (s, 3H), 7.33 – 7.18 (m, 6H), 6.44 (d, 1H), 3.96 (d, 1H), 3.77 – 3.63 (m, 1H), 3.53 – 3.45 (m, 3H), 3.23 – 2.81 (m, 3H).

Boc-FF-NBD: N-(tert-butoxycarbonyl)-L-phenylalanine (Boc-Phe-OH) (450 mg, 1.65 mmol), HBTU (624 mg, 1.65 mmol) and HOBt (225 mg, 1.65 mmol) were dissolved in DMF (5 mL) in a round bottom flask and the mixture was stirred with a magnetic stirrer. N, N-Diisopropylethylamine (DIPEA) (525 µL, 3 mmol), and NH2-F-NBD (590 mg, 1.44 mmol) were added at 1-minute intervals respectively and the reaction mixture was stirred for 24 h at room temperature. The reaction mixture was poured into 100 mL of water. The precipitate was collected by filtration and washed with water. The crude product was dried and further purified via column chromatography to give 403 mg brown solid, which was characterized by NMR. ^{1}H NMR (400 MHz, DMSO-d6) δ 9.34 (s, 1H), 8.56 (d, 1H), 8.24 (d, 1H), 8.11 – 7.97 (m, 1H), 7.30 – 7.06 (m, 11H), 6.91 (d, 1H), 6.43 (d, 1H), 4.48 (d, 1H), 4.26 – 4.05 (m, 1H), 3.47 (s, 2H), 3.11 – 2.78 (m, 4H), 2.72 – 2.55 (m, 2H), 1.32 (d, 9H).

NH_2-F-NBD: The intermediate compound (100 mg) was dissolved in 6 mL of 4 M hydrogen chloride solution in dioxane for protection. After stirring for 3 hours, the solvent was evaporated on a rotary evaporator to give an oily residue. Diethyl ether was added to the flask and the contents were stirred gently. A white precipitate formed and was separated by centrifugation, yielding about 85 mg of a yellow product. The product was characterized by NMR. ^{1}H NMR (400 MHz, DMSO-d6) δ 9.33 (s, 1H), 8.96 (d, J = 8.0 Hz, 1H), 8.54 (d, J = 8.9 Hz, 1H), 8.43 (s, 1H), 8.18 – 8.12 (m, 3H), 7.30 – 7.20 (m, 11H), 6.44 (d, J = 9.0 Hz, 1H), 4.47 (td, J = 8.1, 6.0 Hz, 1H), 4.03 (s, 1H), 3.47 (d, J = 2.3 Hz, 2H), 3.16 – 2.80 (m, 6H). 13C NMR (101 MHz, DMSO-d6) δ 171.31, 168.31, 137.78, 135.19, 130.02, 129.96, 129.61, 129.05, 128.90, 128.63, 127.69, 127.54, 126.89, 66.83, 55.00, 53.67, 53.61, 37.30. MALDI MS(EI): calculated for $C_{26}H_{27}N_7O_5$: 517.2074 (M+); found 518.2239 ($M+H^+$).

1.3 Vesicle production

The preparation of the polymersomes was carried out using a droplet microfluidic technique. The chips used to design the vesicles were prepared as described in the next sections.

1.3.1 Photolithography

Photolithography was chosen as the technique for manufacturing the chips, which consists of transferring geometric shapes on a mask to the surface of a silicon wafer. The first step in the production of the wafers is chemical cleaning, where the wafers are cleaned to remove particles or organic, ionic or metallic impurities. The pre-backing step was carried out in a hot plate at 200oC for 5 minutes, followed by two steps of spin coating: first, with SU-83050 photoresist at 2200 rpm for 30 seconds up to a height of 70 µm; second, with SU-83030 photoresist at 1800 rpm for 30 seconds up to a height of 40 µm. After spin coating, the wafers were again pre-baked on a hot plate at 65 oC for 1 min, and soft-baked at 95 oC for 20 min to remove the solvents from the photoresist coating, followed by cooling at 65 oC for 1 min. The photoresist and wafer were then aligned with a film mask containing the chip designs. The photoresist was exposed to high intensity UV light for 5 seconds through the pattern on the mask. The post-bake and bake steps were performed at 65 oC for 1 minute and 95 oC for 1 minute, respectively. The final step was the hard bake, which cures the photoresist and improves its adhesion to the wafer surface. The unexposed photoresist was dissolved by soaking the wafer in a developer for 10 min. After the final bake, the wafer was washed in isopropanol and dried with nitrogen. The quality of the wafer was checked with a microscope.

1.3.2 PDMS chip production

For chip fabrication, the wafer containing the channels was placed on a glass dish and covered with 5 mm of the PDMS Sylgard 184 kit in a ratio of 9:1 (PDMS:hardener), corresponding to 40 mg of the mixture. The glass dish containing the wafer and the liquid mixed PDMS and hardener solution was degassed using a conventional vacuum pump and chamber until all air bubbles were removed. The wafers were then placed at 80°C for at least 2 hours to enhance molecular cross-linking. At the end of the cross-linking reaction, the microchips were cut out with a scalpel and pierced with a syringe needle to create a connection between the channels and the outside. The PDMS chips were covered with tape to protect the surface from dust until activation in a plasma cleaner. Glass slides, previously cleaned with ethanol and dried, were then activated together with the PDMS chips in a plasma cleaner for 1 min at 20% power. After this step, the active surfaces of the glass slides and PDMS chips were bonded together to create small channels.

1.3.3 Chip coating

The final step in chip fabrication was to coat the outer channels with a poly(vinyl alcohol)-water solution (1 wt%) for 1 min, which was done immediately after activation. This step makes the outer fluid channel hydrophilic and is essential to prevent the center fluid from sticking to the otherwise hydrophobic walls of the outlet channel. The PVA solution was sucked down the outer fluid channel to the chip outlet using a conventional vacuum pump. After the coating step, the chips were baked at 80 °C for 120 min. After fabrication, the quality of the channels was inspected under a microscope.

1.3.4 Polymersome production

All solutions used for vesicle preparation were freshly prepared. The typical inner solution was prepared with 700 µL milli-Q water, 300 µL HEPES (1 M), and 10 mg of the synthesized FFM peptide. Other peptide concentrations (2.5, and 5 mg/mL) were tested, and empty polymersomes (without FFM peptide in the inner fluid) were also produced. The middle solution was prepared by adding 10 mg of PB22-b-PEO14 in 1 mL of oleyl alcohol and kept at 37°C for 1 hour with stirring. The outer solution consisted of 700 µL Milli-Q water and 300 µL NaCl (1 M) solution. The pH during polymersome preparation was 5.5.

All solutions were filled into 1 mL syringes and connected to the microfluidic chip via needle and tubing. Vesicles were collected through a tube placed on the chip outlet connected to an Eppendorf tube. The syringes were attached to the microfluidic pump system and the chip was placed under a microscope objective. The vesicles were ejected until the steady state of double emulsion production was reached. Only when stable vesicle production was achieved was collection initiated for further vesicle analysis. The microfluidic device setup and solution compositions are shown in Table S1.

Table S1. Typical microfluidic setup for polymersome production

Component	Concentration	Solution	Flow rate (µL/h)
HEPES (pH 6) / FFM peptide	300mM / 10 mg/mL	Inner fluid (IF)	40
PB_{22}-b-PEO_{14} in oleyl alcohol	10 mg/mL	Middle fluid (MF)	40
NaCl in milli-Q	300 mM	Outer fluid (OF)	400

After 20 min of vesicle production, 10 µL of vesicles (taken from the bottom of the Eppendorf used for vesicle collection) were placed in a microscope chamber containing 300 µL of NaCl 300 mM solution in 5 mM HEPES and 2 µL of Nile Red solution (1 mg/mL in DMSO) at pH 5.5.

In order to understand the influence of pH on the division events caused by peptide encapsulation, the pH of the chamber solution was adjusted by adding 0.1 M NaOH solution to obtain different pH values: 6.6, 7.7, 8.8, 10.6, and 11.2. Each pH was maintained for 1 hour for video recording. The experiments with the labeled peptide (FFM-NBD) followed the same procedure.

To study the influence of osmotic pressure on vesicle division, experiments were also performed with the chamber filled with FFM peptide (10 mg/mL). In this condition, the stability of the produced polymersomes decreased and the experiments could be performed only up to pH 7.7.

In order to understand the effect of FFM to the preformed polymersomes solution along with the increment of the pH, empty polymersomes (without FFM in the inner fluid) were produced via microfluidics (Table S1) and placed on a microscope chamber containing 300mM NaCl in 5mM HEPES and 10 mg/mL of FFM. Each pH was maintained for 1 hour for video recording.

In the cholesterol experiments, polymersomes were prepared at the concentration and setup shown in Table S2. The typical medium was replaced by a solution containing 10 mg PB_{22}-b-PEO_{14}, 10 mg cholesterol, and 1 mL oleyl alcohol. It was kept under stirring for 1 hour at 37°C.

Table S2. Microfluidics setup for polymersome containing cholesterol on the membrane

Component	Concentration	Solution	Flow rate (µL/h)
HEPES (pH 6)	300mM	Inner fluid (IF)	40
PB_{22}-b-PEO_{14} and cholesterol in oleyl alcohol	10 mg/mL and 10 mg/mL	Middle fluid (MF)	40
NaCl in milli-Q	300 mM	Outer fluid (OF)	400

1.3.5 Liposome production

All solutions used for vesicle preparation were freshly prepared. The inner solution was prepared with 300 mM HEPES (pH 6) and 10 mg/mL FFM peptide. The middle phase is oleyl alcohol. All lipids are stored as a stock solution in chloroform. The lipids are dried in a glass vial for at least 1 hour under a weak nitrogen stream and then the appropriate middle liquid phase is added. To ensure that all lipids are dissolved in the middle fluid phase, the glass vial containing the dried lipids and the middle fluid phase is incubated for at least 2 hours and vortexed several times. The outer solution consisted of 700 µL of Milli-Q water and 300 µL of sucrose solution (1 M). All solutions were filled into 1 mL syringes and connected to the microfluidic chip via needle and tubing. The vesicles were collected through a tube placed on the chip outlet connected to an Eppendorf tube. The syringes were attached to the microfluidic pump system and the chip was placed under a microscope objective. The vesicles were ejected until the steady state of double emulsion production was reached. Only when a stable vesicle production was reached, the collection for further vesicle analysis was initiated. The microfluidic device setup and solution compositions are shown in Table S3.

Table S3. Microfluidics setup for liposome production

Component	Concentration	Solution	Flow rate (µL/h)
HEPES (pH 6) + FFM peptide	300mM / 10 mg/mL	Inner fluid (IF)	100
POPC and cholesterol in oleyl alcohol	10 mg/mL and 2.5 mg/mL	Middle fluid (MF)	40
Sucrose	300 mM	Outer fluid (OF)	400

1.3.6 Microscopy

Video recordings of the effect of pH on the cleavage mechanism were made on a light microscope using LAS X Office software. Frames were acquired every 10 seconds from 0 to 60 minutes. Before each addition of NaOH solution, the pH of the chamber was measured and stabilized for 5 min before the start of image acquisition.
Micrographs of polymersomes were taken on confocal laser scanning microscopy (Leica TCS SP5, Wetzlar, Germany) using a Leica HC PL APO CS2 63x/1.2 water immersion objective in 8-well glass bottomed µ-slides. Images were acquired at a resolution of 512x512 or 1024x1024 at a scanning speed of 400 Hz and analyzed using ImageJ ver. 1.53c software. Nile Red fluorescence was excited at 561 nm and emission was detected by a photomultiplier in the spectral range 550-640 nm. Resorufin fluorescence was excited at 476 nm and the emission was detected by a photomultiplier with a spectral range of 550-640 nm. The labeled FFM peptide (FFM-NBD) was excited at 476 nm and the emission was detected at 539 nm.

1.3.7 Fluorescence Confocal Spectroscopy (FCS) assays

Fluorescence Correlation Spectroscopy experiments were performed on a commercial device, LSM 880 (Carl Zeiss, Jena, Germany). The excitation was done with either a HeNe (543 nm, for the Nile Red fluorophores) or an Argon (488 nm, for the NBD fluorophores) laser focused into the studied samples through a C-Apochromat 40×/1.2 W water immersion objective (Carl Zeiss, Jena, Germany). The emission light was collected with the same objective and after passing through a confocal pinhole, directed to a spectral detection unit (Quasar, Carl Zeiss) in which a detection range of 560 – 700 nm (for 543 nm excitation) or 500 – 550 nm (for 488 excitation) was selected. Eight-well polystyrene chambered cover glasses (Nunc™ Lab-Tek™, Thermo Fisher Scientific, Waltham, MA, USA) were used as sample cells for the studied vesicle dispersions. After sedimentation of the vesicles at the chamber bottom, they were first visualized with the confocal laser scanning mode of the microscope. The confocal observation volume was positioned either inside/outside of the vesicles or precisely over the polymerosome membrane at the “North pole” of a vesicle and series of FCS measurements with a total duration of 150 s were performed. These experiments were repeated multiple times and with different vesicles. The time-dependent fluctuations of the fluorescent intensity δI(τ) were recorded and analyzed by an autocorrelation function $G(\tau) = 1 + \langle\delta I(t) \cdot \delta I(t + \tau)\rangle/\langle I(t)\rangle^2$. The obtained in this way experimental autocorrelation curves were fitted with a theoretical model function [4] for either bulk 3D diffusion (eq. S1) or membrane 2D diffusion (eq. S2)

$$G(\tau) = 1 + \left[1 + \frac{f_T}{1 - f_T} e^{-\tau/\tau_T}\right] \frac{1}{N} \sum_{i=1}^{m} \frac{f_i}{\left[1 + \frac{\tau}{\tau_{Di}}\right] \sqrt{1 + \frac{\tau}{S^2 \tau_{Di}}}} \quad \text{(S1)}$$

$$G(\tau) = 1 + \left[1 + \frac{f_T}{1 - f_T} e^{-\tau/\tau_T}\right] \frac{1}{N} \sum_{i=1}^{m} \frac{f_i}{\left[1 + \frac{\tau}{\tau_{Di}}\right]} \quad \text{(S2)}$$

Here, N is the average number of diffusing fluorescence species in the observation volume, f_T and τ_T are the fraction and the decay time of the triplet state, τ_{Di} is the diffusion time of the *i-th* diffusion component and S is the so-called structure parameter, $S = z_0/r_0$, where z_0 and r_0 represent the axial and radial dimensions of the confocal volume, respectively. Furthermore, the diffusion time, τ_{Di}, is related to the respective diffusion coefficient, D_i, through: $\tau_{Di} = \frac{r_0^2}{4Di}$. The fits yielded the corresponding diffusion times, and subsequently the diffusion coefficients of the studied fluorescent species. As the value of r_0 depends on the specific characteristics of the optical setup, calibration experiments were performed using fluorescent tracers with known diffusion coefficients, i.e. Alexa Fluor 488 and Alexa Fluor 546. Two different sets of FCS experiments were performed.

The first set was done to investigate the diffusion of external species in the polymersome membrane, in the presence or absence of FFM peptide encapsulated in the polymersome. The microscope chamber well was prepared with 300 µL of a 300 mM NaCl solution containing 5 mM HEPES (pH 5.5), and the pH was gradually increased by the addition of NaOH (0.1M) solution to reach the pHs 8.8 and 11.2. The diffusion of Nile Red in the polymersome membrane was studied by adding 2µL of this dye (1µg/mL in DMSO) in the chamber. 10µL of dispersion containing empty or loaded vesicles (containing 10 mg/mL of FFM peptide) were placed into the chamber well, and the diffusion time of Nile Red at the polymersome membrane was estimated in three different pHs (5.5, 8.8, and 11.2). NileThe recorded experimental autocorrelation curves were fitted with eq. S2 using one diffusing component (*m* = 1).

The second set of experiments was done to investigate the diffusion behavior of the labeled FFM peptide (FFM-NBD) inside and outside the polymersome and at the polymersome membrane along with the pH increase. 300 µL of a 300 mM NaCl solution containing 5mM HEPES (pH 5.5) along with 10µL of polymersomes loaded with FFM peptide (95 wt% FFM + 5 wt% FFM-NBD) were placed into the microscope chamber well. The pH was gradually increased by the addition of NaOH (0.1M) solution to reach the pHs 8.8 and 11.2. The experimental autocorrelation curves recorded outside the polymerosomes were fitted with eq. S1 using one diffusing component (*m* = 1). The autocorrelation curves recorded inside the polymerosomes were fitted with eq. S1 using two diffusing components (*m* = 2). This was necessary to account for the very large aggregates that were present inside the polymerosomes in addition to the individually diffusing FFM-NBD molecules. In order to improve the fits stability, the fast diffusing component’s diffusion time was fixed to the respective value of the individual FFM-NBD molecules at the corresponding pH as measured outside the polymerosomes.

Fitting the curves measured for FF-NBD in the polymerosome membrane should be done with great care. Indeed, the FCS confocal volume with its normal dimension of ~ 1 µm covers not only the membrane, but also parts of the inside and outside of the polymerosome. Thus, in addition to the 2D diffusion of the membrane bound FF-NBD, the FCS autocorrelation curve may also reflect “bulk”, 3D diffusion of the FF-NBD. To account for this effect, two-component fits, in which one of the components was fixed to the “bulk” 3D diffusion time of the FF-NBD as measured outside the polymerosomes were attempted by combining eqs. S1 and S2. However, the fits showed only very minor fraction of this component and the 2D diffusion time for the bounded FF-NBD did not change significantly compared to single component fits. Thus, one-component fits with eq. S2 were chosen as most appropriate. The fact that the obtained diffusion times were significantly lower than those measured for Nile Red in the

polymerosome membrane, indicates that in contrast to Nile Red, the FF-NBD are not well incorporated in the hydrophobic part of the vesicle wall bi-layer. Most likely while some the FF-NBD are constantly adsorbing/desorbing to the membrane with adsorption time so short that multiple adsorption/desorption events may occur during the passage of an individual FF-NBD molecule through the FCS confocal observation volume. As shown earlier[5] in such cases FCS cannot distinguish between the two states (bound and unbound) and yields an average diffusion time.

1.3.8 Plate reader measurements

The influence of pH on the fluorescence intensity of labeled FFM peptide (FFM-NBD) was determined using an infinite plate reader (Tecan), where the fluorescence intensity was determined at emission and excitation wavelengths of 539 nm and 467 nm, respectively. Aqueous solutions containing 2 mg/mL FFM-NBD were placed in a black 96-well plate with a transparent bottom at four different pHs (3, 5.5, 8.8, and 11.2). All measurements were performed in triplicate.

1.4 Determination of the isoelectric point of FFM peptide

The isoelectric point of the phenylalanine-phenylalanine-methionine peptide, which corresponds to a net charge of zero, was calculated from the pKa values of the α-carboxyl group, α-ammonium ion, and side chain for each amino acid, as shown in Table S4.

Table S4. pKa values of amino acids

Amino acid	pKa1 (α-carboxyl group)	pKa2 (α-ammonium ion)	pKa3 (side chain)
Phenylalanine	1.83	9.13	-
Methionine	2.28	9.21	-

Since the peptide bonds are not ionizable and the amino acids phenylalanine and methionine do not have an ionizable side chain, the net charge of the peptide will be zero between the pH values corresponding to the lowest pKa (2.28, corresponding to the α-carboxyl group of methionine) and the highest (9.13, corresponding to the α-ammonium of phenylalanine). In this sense, the isoelectric point of the FFM peptide is the average between these two values (5.71). From 0 to the pH equal to the isoelectric point, the peptide is fully protonated (net charge = 1). Beyond this pH, it is deprotonated (net charge = -1).

1.5 Statistical analysis – Influence of osmotic pressure and pH in polymersome division

1.5.1 Influence of pH

The division yield (DY) was determined by image analysis of bright field microscopy micrographs. DY was calculated as A/B, where B represents the total number of first generation vesicles in an image and A represents the number of vesicles that had one or more daughter vesicles. It's important to note that DY is based on the number of first-generation vesicles producing daughter vesicles, not the number of daughter vesicles formed (during a 1-hour observation). This approach is taken because the process is highly dynamic, making it difficult to reliably determine the total number of daughter vesicles. Therefore, DY measures the probability of first-generation vesicles to divide under different conditions, but it does not predict the number of daughter vesicles that will be produced.

The vesicles were maintained at the pH for 1h, and then the pH was gradually increased by adding NaOH solution directly into the microscope chamber. Each experiment was performed in triplicate. ANOVA and Tuckey-Kramer tests were performed to compare the effect of different pH on the number of division events. The least significant difference (LSD) between samples was calculated according to equation 3.

$$LSD = t_{0.05} * \sqrt{\left(\frac{1}{n_i} + \frac{1}{n_j}\right)\frac{MSW}{2}} \quad (3)$$

Where:

t = critical value determined using a table with α = 0.05 and degrees of freedom (12). In this case, it was 1.782.

MSW = Mean Square Within, obtained from the results of the ANOVA test.

n = number of scores used to calculate the means.

If the LSD was greater than the absolute difference between the two pH values compared, then it is possible to ensure that there is a significant difference in the number of division events between the two pH values compared.
To determine the effect of pH on the number of daughter vesicles produced per mother vesicle, the number of daughter vesicles produced per vesicle during division was estimated manually in triplicate. The results of the statistical analysis regarding the influence of pH on the division events are shown in Tables S6 - S9.

1.5.2. Influence of osmotic pressure

In experiments where the peptide concentration was the same inside and outside the vesicles, the Division Yield (DY) and the number of daughter vesicles produced per mother vesicle were estimated, according to the method presented in Section 1.5.1 All the results were compared to the experiments with the chamber without peptide (with high difference in osmotic pressure inside and outside the polymersomes). The estimation was carried out from pHs 5.5 to 7.7, once beyond this range, no more vesicles were found in the chamber due to their collapse. The number of division events and number of daughter vesicles per mother vesicle in these experiments were shown in Tables S6 and S9. ANOVA tests performed to estimate the significance of osmotic pressure affecting the number of division events were shown in Tables S10 and S14.

1.6 Enzymatic cascade reaction inside the polymersomes

Vesicle formation for experiments in which the enzymatic cascade reaction was performed inside the polymersomes followed the composition and setup shown in Table S5.

Table S5. Microfluidic setup for experiments involving the enzymatic cascade reaction for resorufin production.

Component	Volume/Concentration	Solution	Flow rate (µL/h)
Milli-Q water	43.5 µL		
FFM solution (20 mg/mL)	250 µL		
HEPES 1M	150 µL		
Glucose 1M	50 µL	Inner fluid (IF)	40
HPR 1mg/mL in PBS	1.5 µL		
GOx 5mg/mL in PBS	5 µL		
PB22-b-PEO14 in Oleyl alcohol	10 mg/mL	Middle fluid (MF)	40
NaCl in milli-Q	300 mM	Outer fluid (OF)	400

For these experiments, 10 µL of vesicles were taken into a chamber containing 300 µL of 400 mM NaCl solution + 1 µL Amplex Red (1mM). The formation of the fluorescent product was analyzed using confocal laser microscopy (Leica TCS SP5, Wetzlar, Germany) using a Leica HC PL APO CS2 63x/1.2 water immersion objective in µ-slide 8 wells with a glass bottom. The images were recorded at a 512x512 or 1024x1024 resolution at a 400 Hz scan speed and analyzed using ImageJ ver. 1.53c software. Resorufin fluorescence was excited at 571 nm and the emission was detected by a photomultiplier using a bandwidth of 550-640 nm.

The cascade reaction was also performed in bulk to assess its viability. The formation of the resorufin product was attested by the fluorescence emission at 584nm, measured using a TECAN M1000 microplate reader. A standard calibration curve of fluorescence intensity versus resorufin concentration was included as Figure S7 in Supporting Information.

1.7 Evaluating the behavior of FF-NBD peptide in bulk

To evaluate the self-assembly of the FF-NBD peptide in bulk along with the pH increase, a microscope chamber was prepared with 300 mM NaCl in 5mM HEPES at different pHs (5.5, 6.6, 7.7, 8.8, 10.6 and 11.2). FFM+FF-NBD (95:5) was added to the system at three different concentrations: 2.5, 5, and 10 mg/mL. Each pH was maintained for 30 minutes and micrographs were taken using CLSM.

2 Statistical analyses of the influence of pH and osmolarity on polymersome division

2.1 Statistical analyses of the pH influence in the division yield (DY)

Table 6 shows the division yield (DY) measured at different pHs. The division yield represents the number of vesicles that divided per 100 vesicles counted. All division experiments were performed in triplicate to further estimate an average number of division events at each pH and a standard deviation.

Table S6. Division yield (DY) of polymersomes investigated in the pH range of 5.5 to 11.2.

	pH 5.5	pH 6.6	pH 7.7	pH 8.8	pH 10.6	pH 11.2
First experiment	0.054	0.116	0.2	0.4	0.1	0.083
Second experiment	0.039	0.082	0.125	0.5	0.286	0.167
Third experiment	0.04	0.103	0.214	0.2	0.108	0.182
DY Average	**0.044**	**0.100**	**0.180**	**0.367**	**0.165**	**0.144**
DY St. Deviation	0.007	0.014	0.039	0.125	0.086	0.043

From the data presented in Table 6, an estimation of significant differences within the division yield (DY) within the investigated pH range was required, which led to the application of an ANOVA test (Table S7). The p-value between the division yield at different pHs was less than 0.05, indicating that there was a significant difference in DY between the pHs tested.

Table S7. ANOVA test for the effect of pH on the division yield (DY)

Source Of Variation	SS	DF	MF	F	P-Value	F-Crit
Between Groups	0.180	5	0.036	5.429	0.008	3.106
Within Groups	0.080	12	0.007			
Total	0.260	17				

SS: sum of squares due to the source; DF: degrees of freedom; MF: the mean of squares due to the source; F: F-statistic; F-Crit: F-critical.

The ANOVA test itself is not able to indicate where the significant differences are in this case. That is, from Table S7 we only know that there is a significant difference between the DYs among the pHs evaluated. For this reason, a Tuckey-Kramer test was used to compare the DY averages of each pH to determine in which ranges there is a significant difference. The results are presented in Table S8. LSD values were calculated using Equation 1 and represent a significant difference if they are greater than the absolute difference between the two compared means (Abs med). Significant differences are highlighted in red in Table S8.

Table S8. Tuckey-Kramer test for the effect of pH on vesicle division

Comparisons	Average 1	Average 2	Abs med	LSD
pH 5.5 - pH 6.6	0.044306529	0.100261136	0.055954607	0.083829509
pH 5.5 - pH 7.7	0.044306529	0.179761905	0.135455376	0.083829509
pH 5.5 - pH 8.8	0.044306529	0.366666667	0.322360138	0.083829509
pH 5.5 - pH 10.6	0.044306529	0.164607465	0.120300936	0.083829509
pH 5.5 - pH 11.2	0.044306529	0.143939394	0.099632865	0.083829509
Comparisons	Average 1	Average 2	Abs med	LSD
pH 6.6 - pH 5.5	0.100261136	0.044306529	0.055954607	0.083829509
pH 6.6 - pH 7.7	0.100261136	0.179761905	0.079500769	0.083829509
pH 6.6 - pH 8.8	0.100261136	0.366666667	0.266405531	0.083829509
pH 6.6- pH 10.6	0.100261136	0.164607465	0.064346329	0.083829509
pH 6.6 - pH 11.2	0.100261136	0.143939394	0.043678258	0.083829509
Comparisons	Average 1	Average 2	Abs med	LSD
pH 7.7 - pH 5.5	0.179761905	0.044306529	0.135455376	0.083829509
pH 7.7 - pH 6.6	0.179761905	0.100261136	0.079500769	0.083829509
pH 7.7 - pH 8.8	0.179761905	0.366666667	0.186904762	0.083829509
pH 7.7 - pH 10.6	0.179761905	0.164607465	0.01515444	0.083829509
pH 7.7 - pH 11.2	0.179761905	0.143939394	0.035822511	0.083829509
Comparisons	Average 1	Average 2	Abs med	LSD
pH 8.8 - pH 5.5	0.366666667	0.044306529	0.322360138	0.083829509
pH 8.8 - pH 6.6	0.366666667	0.100261136	0.266405531	0.083829509
pH 8.8 - pH 7.7	0.366666667	0.179761905	0.186904762	0.083829509
pH 8.8 - pH 10.6	0.366666667	0.164607465	0.202059202	0.083829509
pH 8.8 - pH 11.2	0.366666667	0.143939394	0.222727273	0.083829509
Comparisons	Average 1	Average 2	Abs med	LSD

pH 10.6 - pH 5.5	0.164607465	0.044306529	0.120300936	0.083829509
pH 10.6 - pH 6.6	0.164607465	0.100261136	0.064346329	0.083829509
pH 10.6 - pH 7.7	0.164607465	0.179761905	0.01515444	0.083829509
pH 10.6 - pH 8.8	0.164607465	0.366666667	0.202059202	0.083829509
pH 10.6 - pH 11.2	0.164607465	0.143939394	0.020668071	0.083829509
Comparisons	Average 1	Average 2	Abs med	LSD
pH 11.2 - pH 5.5	0.143939394	0.044306529	0.099632865	0.083829509
pH 11.2 - pH 6.6	0.143939394	0.100261136	0.043678258	0.083829509
pH 11.2 - pH 7.7	0.143939394	0.179761905	0.035822511	0.083829509
pH 11.2 - pH 8.8	0.143939394	0.366666667	0.222727273	0.083829509
pH 11.2 - pH 10.6	0.143939394	0.164607465	0.020668071	0.083829509

LSD: Least significant difference

From the analysis of Table S8, the following can be concluded:

Increasing the pH from 5.5 to 7.7, 8.8, 10.6, or 11.2 significantly increased the division yield.
Increasing pH from 6.6 to 8.8 significantly increased division yield.
Increasing pH from 7.7 to 8.8 significantly increased division yield.
Increasing the pH from 8.8 to 10.6 or 11.2 significantly decreased the division yield.

2.2 Statistical analyses of the effect of pH on the number of daughter vesicles produced per mother vesicle

To determine the effect of pH on the number of daughter vesicles produced by each mother vesicle, we first estimated the total number of vesicles in the chamber using a confocal microscope. Then we estimated division yield (YD), and finally we estimated the number of daughter vesicles produced by each divided vesicle. The number of daughter vesicles produced at each pH was determined as the average number of daughter vesicles produced per parent vesicle at each pH. These results are presented in Table S9. Experiments were performed in triplicate to determine the average number and variance.

Table S9. Number of daughter vesicles per mother vesicle at each pH

	First	Second	Third	*Average*	*Variance*
pH 5.5	3.55	3.5	3.83	3.626	0.032675
pH 6.6	3.14	2.42	2.33	2.631	0.198271
pH 7.7	6.5	3.75	3.83	4.694	2.446759
pH 8.8	3.33	2.63	1.5	2.486	0.854745
pH 10.6	5	1.75	1.75	2.833	3.520833
pH 11.2	4	2	2.5	2.833	1.083333

Similar to the division yield, an ANOVA test was performed to determine if pH had a significant effect on the number of daughter vesicles produced per mother vesicle, as shown in Table S10.

Table S10. ANOVA test for the influence of pH on the number of daughter vesicles produced per mother vesicle at each pH

Source of Variation	SS	df	MF	F	P-Value	F-Crit
Between Groups	10.54765	5	2.11	1.55	0.24	3.106
Within Groups	16.2732	12	1.36			
Total	26.82	17				

SS: sum of squares due to the source; DF: degrees of freedom; MF: the mean of squares due to the source; F: F-statistic; F-Crit: F-critical.

From the analysis of Table S10, it can be concluded that there are no significant differences between the number of daughter vesicles produced per mother vesicle among all the pH values investigated when the p-value is higher than 0.05. In other words, it means that the increase in pH did not cause any effect on the number of daughter vesicles produced per mother vesicle.

2.3 Statistical analyses of the influence of osmolarity in the division yield (DY)

Similarly to Section 1, the influence of osmotic pressure on the DY was studied. The experiments involving the mitigation of osmotic pressure difference outside and inside the polymersomes loaded with FFM were performed by crowding the microscope chamber with FFM. FFM was added to the chamber at the same concentration it had inside the polymersomes (10 mg/mL).

Equaling the concentration inside and outside the polymersomes is expected to prevent the peptide from spreading out by diffusion through the permeable membrane.

The experiments were performed in triplicate, from pH 5.5 to 7.7. It is worth mentioning that the maximum pH for these experiments was 7.7, once beyond this pH the polymersomes were no longer stable. Following the same procedure from the previous section, the number of division events in each pH was determined. The yield of the division was estimated by the number of division events occurring per 100 vesicles counted. The DY from the triplicates were compared with the experiments without FFM in the chamber, and the results are shown in Table S11.

Table S11. Comparison of the division yield (DY) for the chamber with and without peptide in the outer phase.

Group	pH 5.5	pH 6.6	pH 7.7
Chamber without FFM	0.054187	0.115702	0.2
	0.038732	0.081633	0.125
	0.04	0.103448	0.214286
Chamber with 10 mg/mL FFM	0.084906	0.274336	0.4
	0.267717	0.285714	0.274809
	0.182927	0.357143	0.267974

To determine if there was a significant difference in division yield between the experiments with and without FFM in the chamber, an ANOVA test was performed to compare the means between the two different experiments (Table S12). The test yielded p-values less than 0.05 for pH 6.6 and 7.7 (highlighted in red). This indicates a significant difference in division yield between the experiments with and without FFM in the chamber at these pH levels.

In other words, it can be concluded that by reducing the osmotic pressure difference inside and outside the polymersomes by equalizing the FFM concentration, the peptide is forced to remain inside the polymersome instead of diffusing through the permeable membrane. This local concentration significantly increases the number of fission events observed in the experiments, as FFM experiences more frustrated attempts to assemble inside the polymersomes.

Thus, when we compared the division yield in the chamber with and without FFM at pH 6.6, we observed a significantly higher division yield in experiments with peptide also presente in the outer medium. The same behavior was observed at pH 7.7. In conclusion, the division mechanism induced by FFM encapsulation is significantly enhanced by decreasing osmotic pressure differences inside and outside the polymersomes.

Table S12. ANOVA test for pH 5.5, 6.6, and 7.7 comparing the influence of osmotic pressure on the division yield.

pH 5.5	SS	df	MF	F	P-Value	F-Crit
Between Groups	0.027	1	0.027	6.4	0.0646	7.71
Within Groups	0.017	4	0.004			
Total	0.044	5				
**pH 6.6**	**SS**	**df**	**MF**	**F**	**P-Value**	**F-Crit**
Between Groups	0.063	1	0.063	54.77	0.001	7.71
Within Groups	0.004	4	0.001			
Total	0.044	5				
**pH 7.7**	**SS**	**df**	**MF**	**F**	**P-Value**	**F-Crit**
Between Groups	0.020	1	0.020	9.71	0.036	7.71
Within Groups	0.008	4	0.002			
Total	0.044	5				

SS: sum of squares due to the source; DF: degrees of freedom; MF: the mean of squares due to the source; F: F-statistic; F-Crit: F-critical.

2.4 Statistical analyses of the effect of osmolarity on the number of daughter vesicles produced per mother vesicle

Similar to section 2.3, the number of daughter vesicles produced per mother vesicle was studied under the influence of osmolarity. The crowded chamber experiments were performed in triplicate. The number of daughter vesicles in these experiments was estimated as the average of the number of daughter vesicles produced per mother vesicle divided by the number of mother vesicles produced at each pH. The results were compared with those shown in Table S9, and the summary is shown in Table S13.

Table S13. Number of daughter vesicles produced per mother vesicle at pH 5.5, 6.6, and 7.7 comparing the influence of osmotic pressure.

Group		First	Second	Third	*Average*	*Variance*
Outer solution without FFM	pH 5.5	3.55	3.5	3.83	3.626263	0.032675
	pH 6.6	3.14	2.42	2.33	2.630952	0.198271
	pH 7.7	6.5	3.75	3.83	4.694444	2.446759
Outer solution with 10 mg/mL FFM	pH 5.5	6.22	6.85	5.40	6.15838	0.530815
	pH 6.6	6.58	7.97	6.53	7.03104	0.67475
	pH 7.7	7.52	5.13	8.04	6.9038	2.40415

An ANOVA test was performed to determine if there was a significant difference in the number of daughter vesicles produced per mother vesicle between the experiments with and without FFM in the chamber. Results are shown in Table S14. From this test, it is possible to observe p-values lower than 0.05 for pH 5.5 and 6.6 (highlighted in red). Thus, there is a significant difference in the number of daughter vesicles produced per mother vesicle in the experiments with and without the peptide in the outer solution at pH 5.5. The same is observed at pH 6.6. In conclusion, the ANOVA indicates that by decreasing the osmotic pressure difference inside and outside the polymersomes through the equalization of FFM concentration, there is a significant increase in the number of daughter vesicles produced per mother vesicle at pH 5.5 and 6.6.

Table S14. ANOVA test for pH 5.5, 6.6, and 7.7 comparing the effect of osmotic pressure on the number of daughter vesicles per mother vesicle

pH 5.5	SS	df	MF	F	P-Value	F-Crit
Between Groups	9.62	1	9.62	34.135	0.0043	7.71
Within Groups	1.127	4	0.282			
Total	10.74	5				
**pH 6.6**	**SS**	**df**	**MF**	**F**	**P-Value**	**F-Crit**
Between Groups	29.04	1	29.04	66.53	0.0012	7.71
Within Groups	1.75	4	0.437			
Total	37.79	5				
**pH 7.7**	**SS**	**df**	**MF**	**F**	**P-Value**	**F-Crit**
Between Groups	7.32	1	7.32	3.018	0.157	7.71
Within Groups	9.7	4	2.46			
Total	0.044	5				

SS: sum of squares due to the source; DF: degrees of freedom; MF: the mean of squares due to the source; F: F-statistic; F-Crit: F-critical.

Supplementary figures

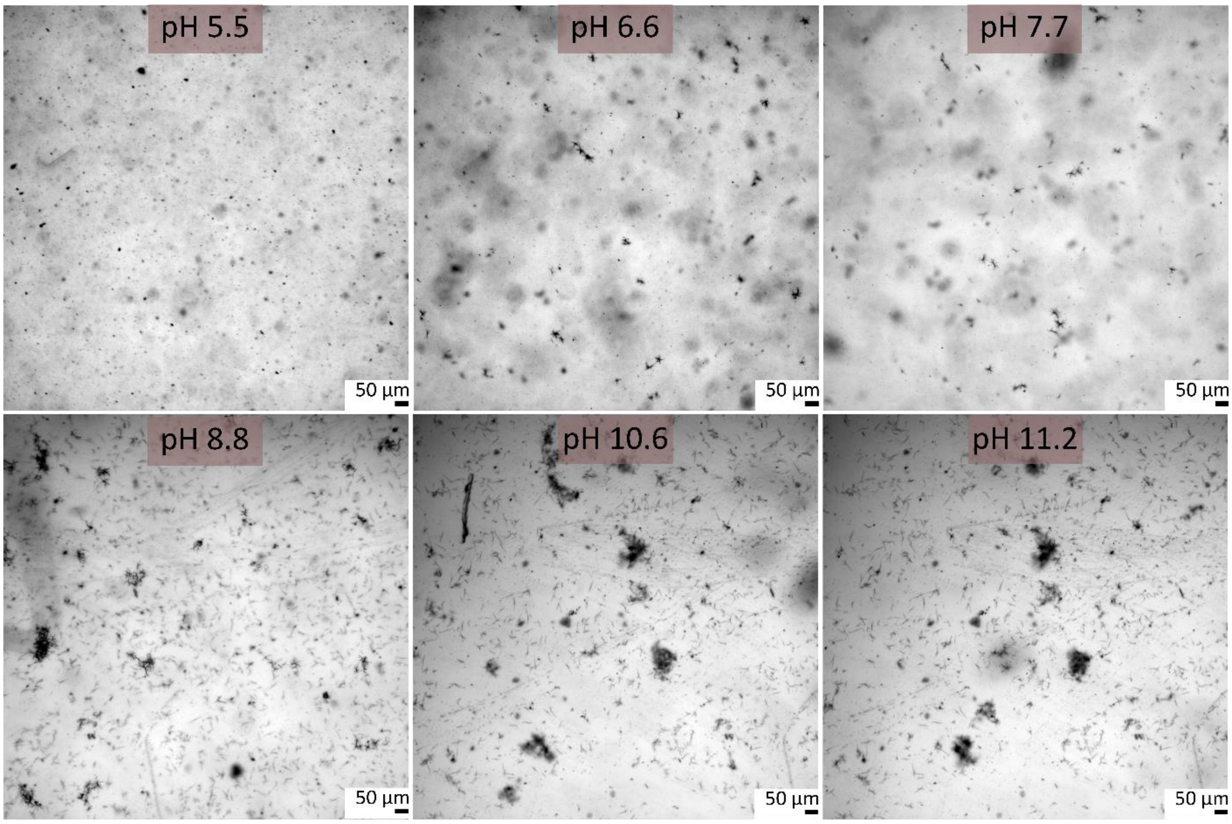

Figure S1. The behavior of FFM peptide (10 mg/mL) in bulk (300 mM NaCl in 5mM HEPES) at different pH. Scale bar = 50 µm.

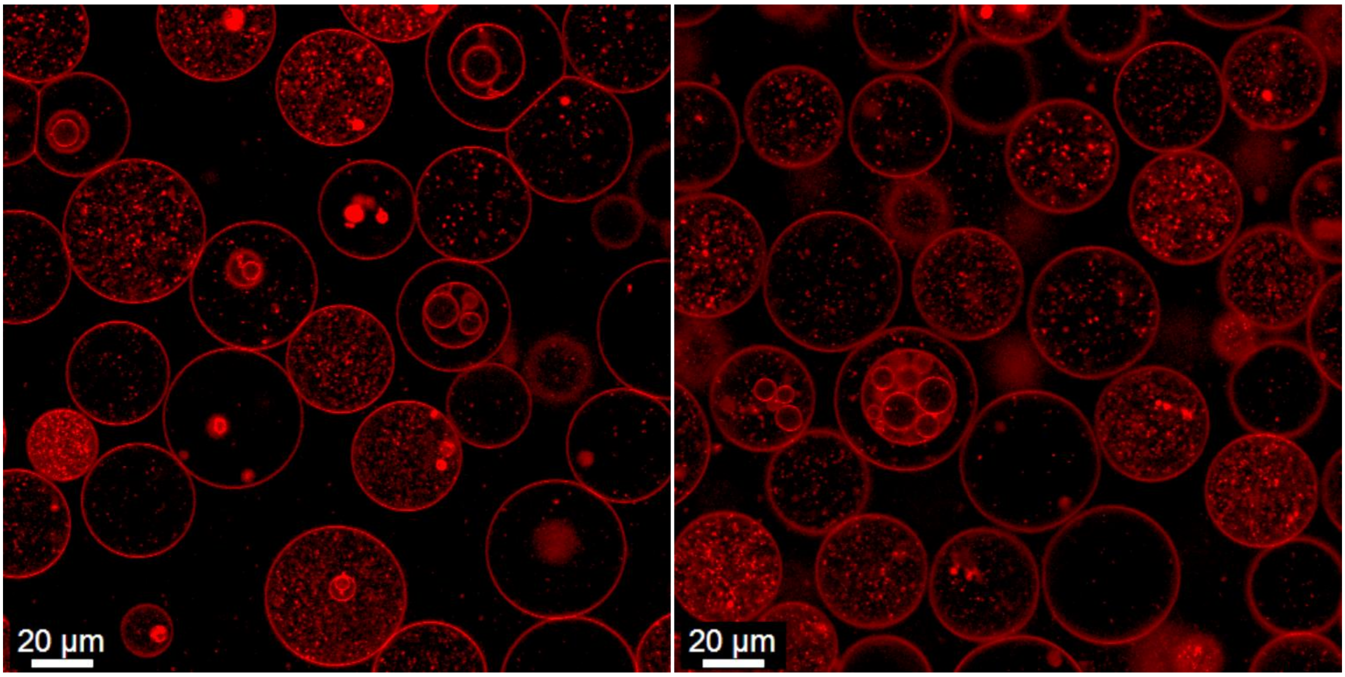

Figure S2. CLSM microphotographs of polymersome division occasioned by the encapsulation of FFM peptide (10 mg/mL) at pH 7.7. Nile Red was used to stain the polymersome membrane. Scale bar = 20 µm.

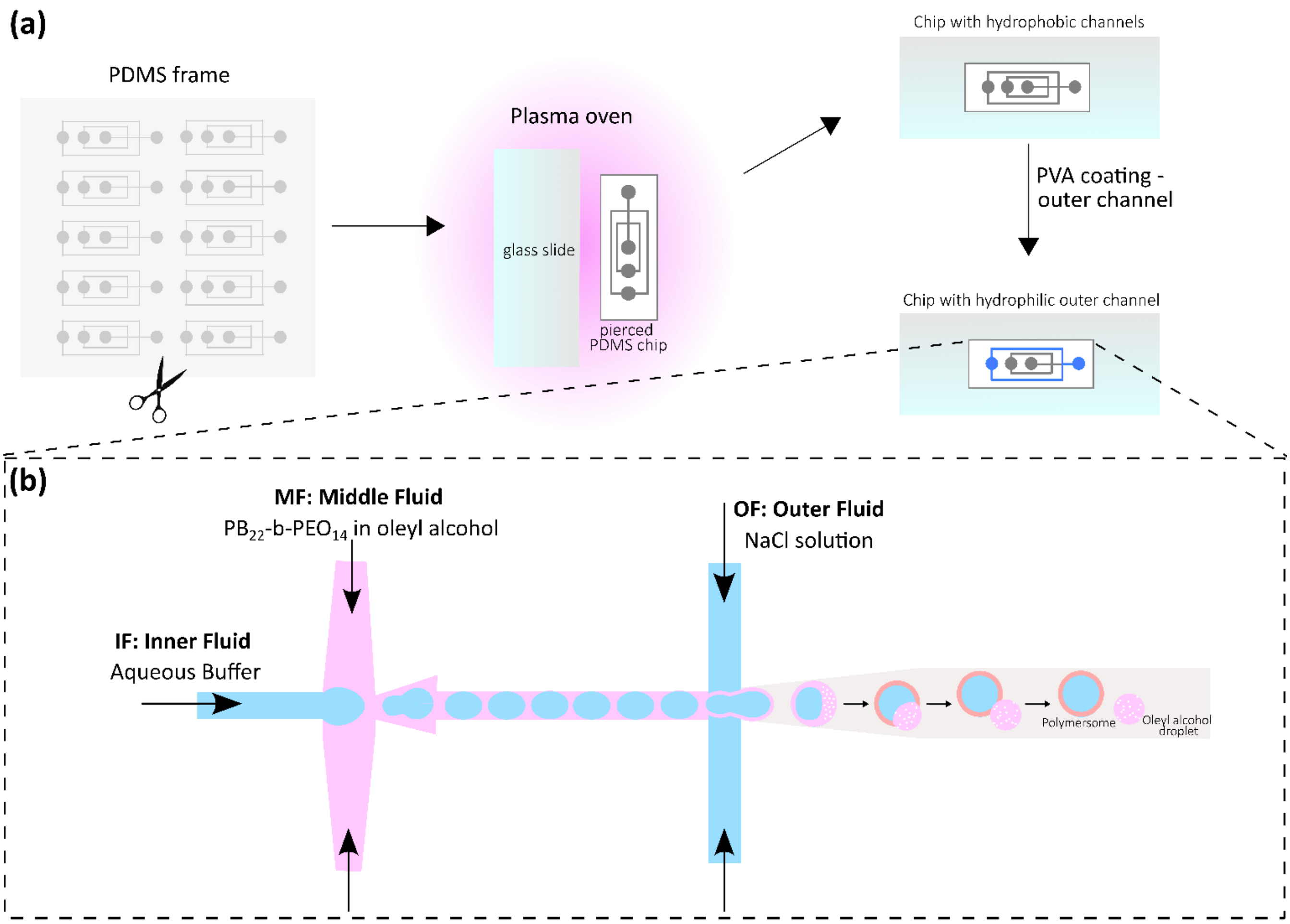

Figure S3. Schematic diagram of microfluidics technique used to prepare polymersomes and liposomes.

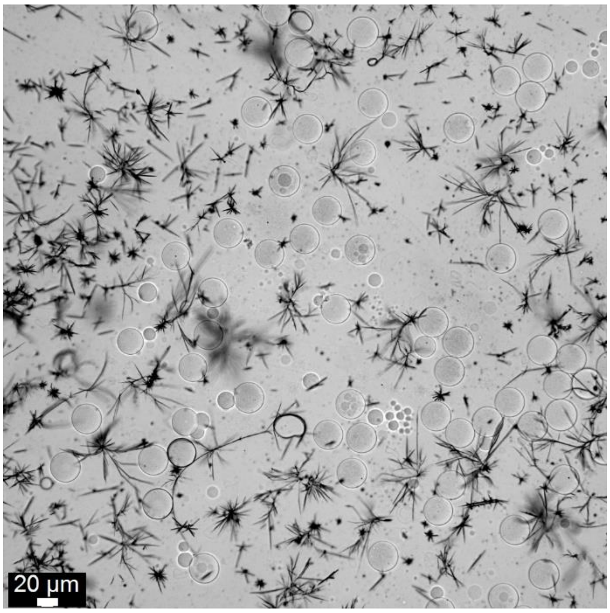

Figure S4. Microfluidics-generated polymersomes containing 10 mg/mL of FFM peptide encapsulated in the vesicle core. The image shows polymersome self-division and FFM self-assembly into fibers outside the polymersomes at pH 7.7. Scale bar = 20 µm.

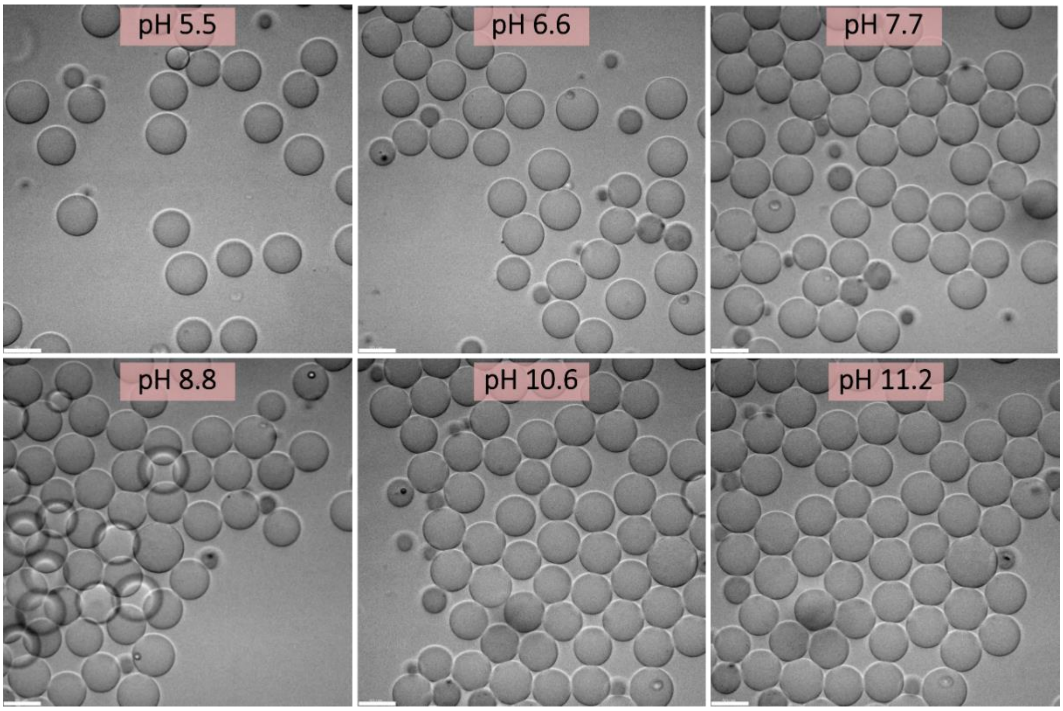

Figure S5. Polymersomes are produced via microfluidics in the absence of FFM peptide in the inner fluid. No division was detected. Scale bar = 50 µm.

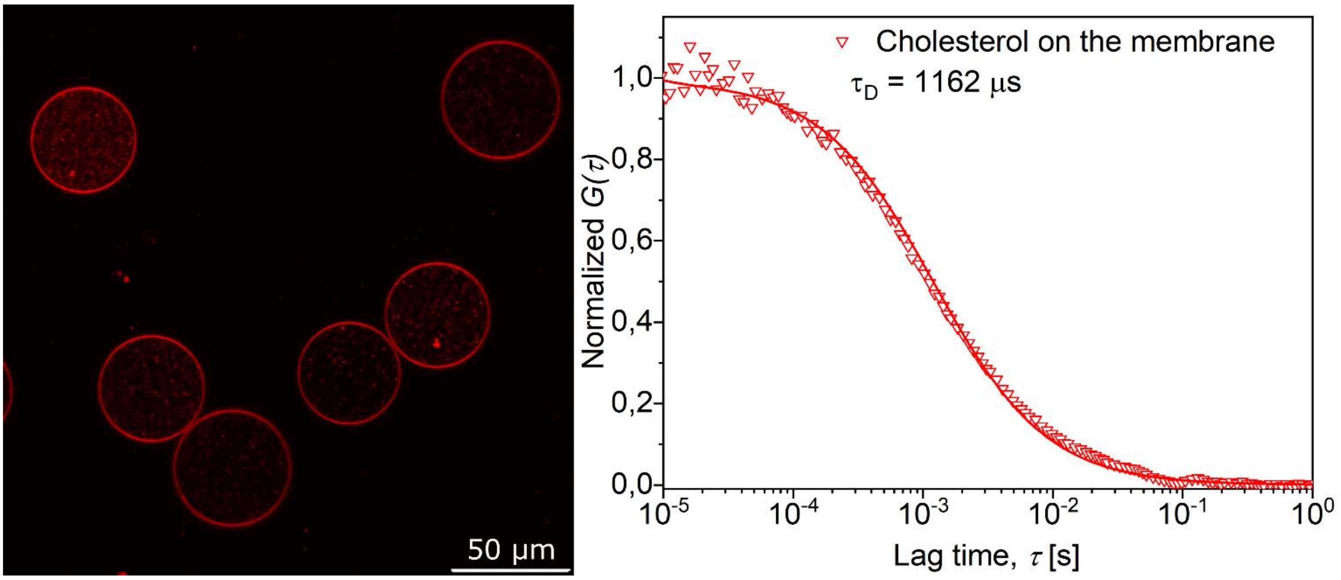

Figure S6. The effect of cholesterol on the diffusion of Nile Red in the polymersome membrane. Measured by Fluorescence Correlation Spectroscopy (FCS). Analysis was performed at pH 8.8. Scale bar = 50 µm.

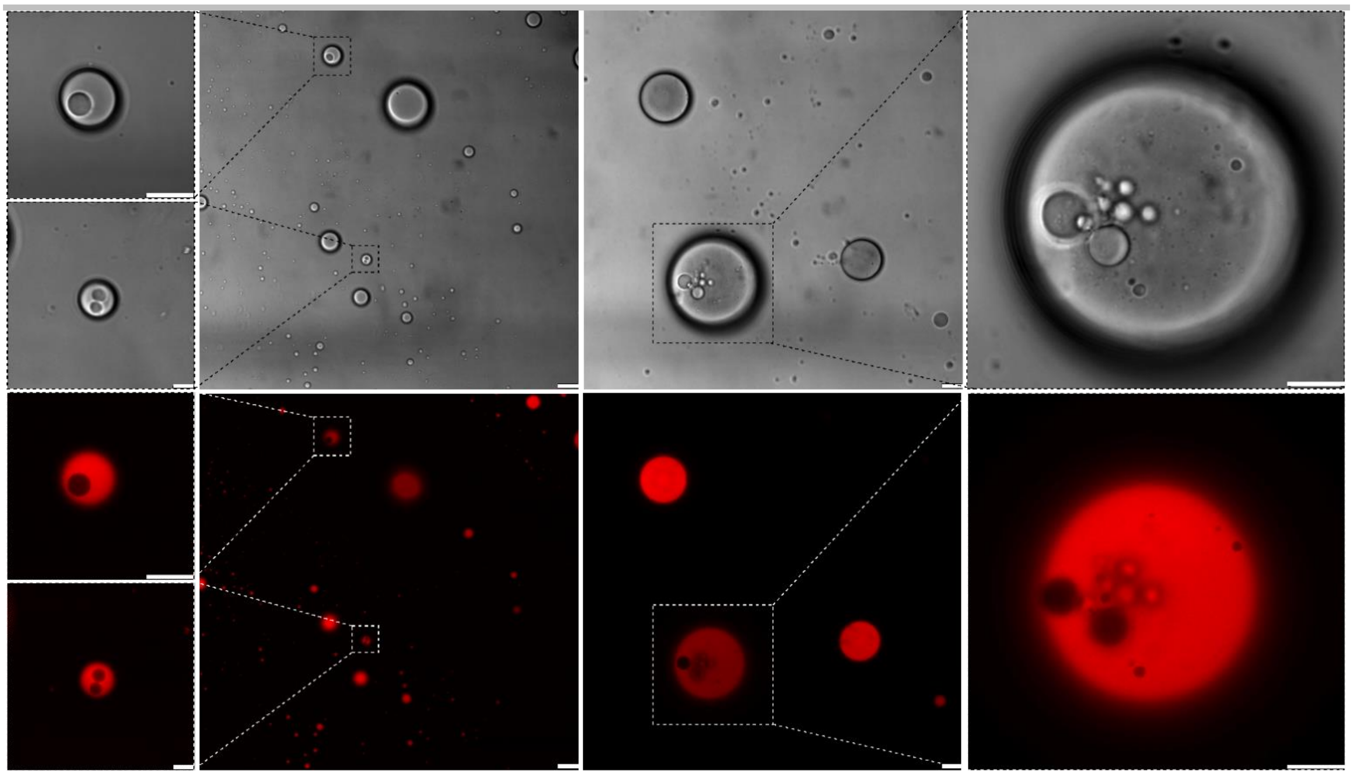

Figure S7. Liposome division induced by encapsulation of 10 mg/mL of FFM peptide at pH 7.7. Scale bar = 10 µm. Liposomes were produced using the microfluidics method and transferred to a microscope chamber containing a solution of 300 mM HEPES and Nile Red (pH 7.7). Micrographs were taken in the first minutes of analysis.

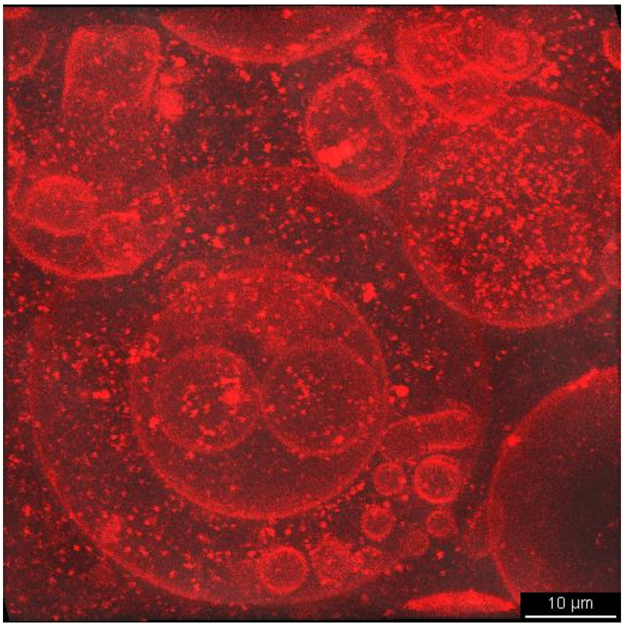

Figure S8. 3D image of a divided polymersome after encapsulation of FFM peptide, at pH 7.7.

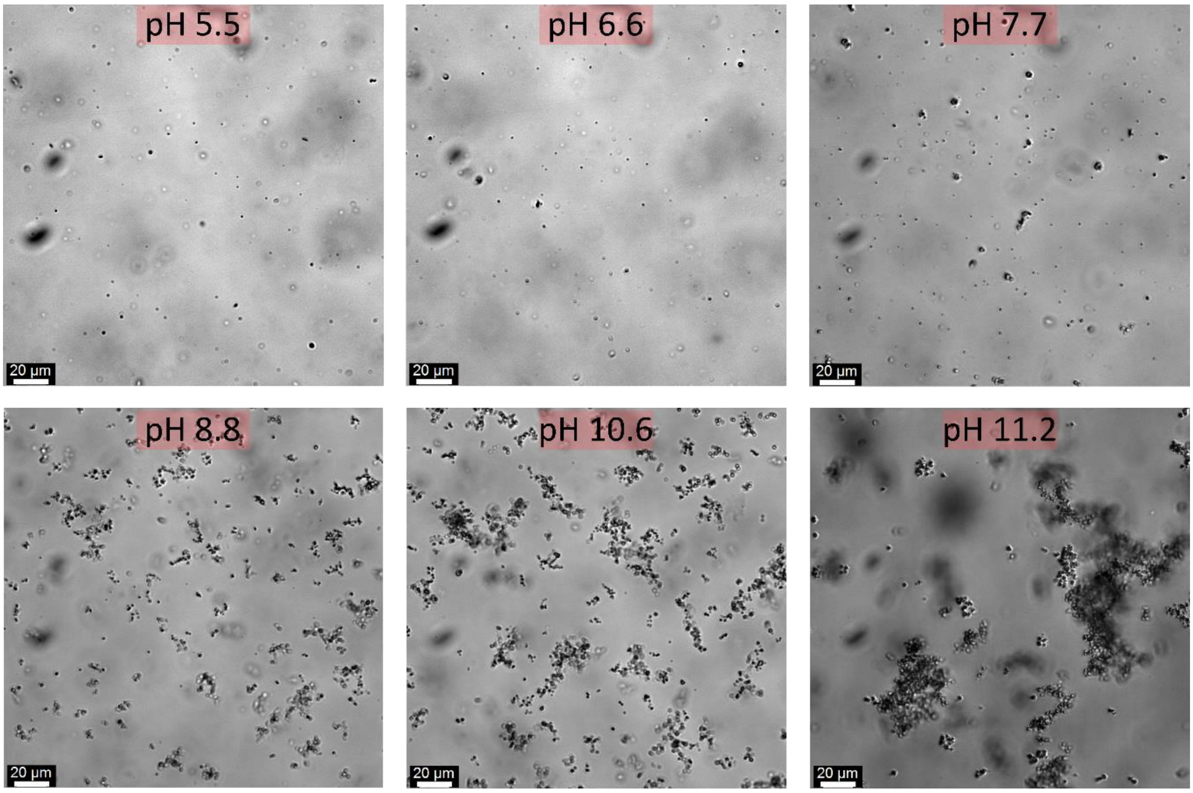

Figure S9. Behavior of FF-NBD peptide (10 mg/mL) in bulk (300 mM NaCl in 5mM HEPES) at different pH values. Bright field microscopy images. Scale bar = 20 µm.

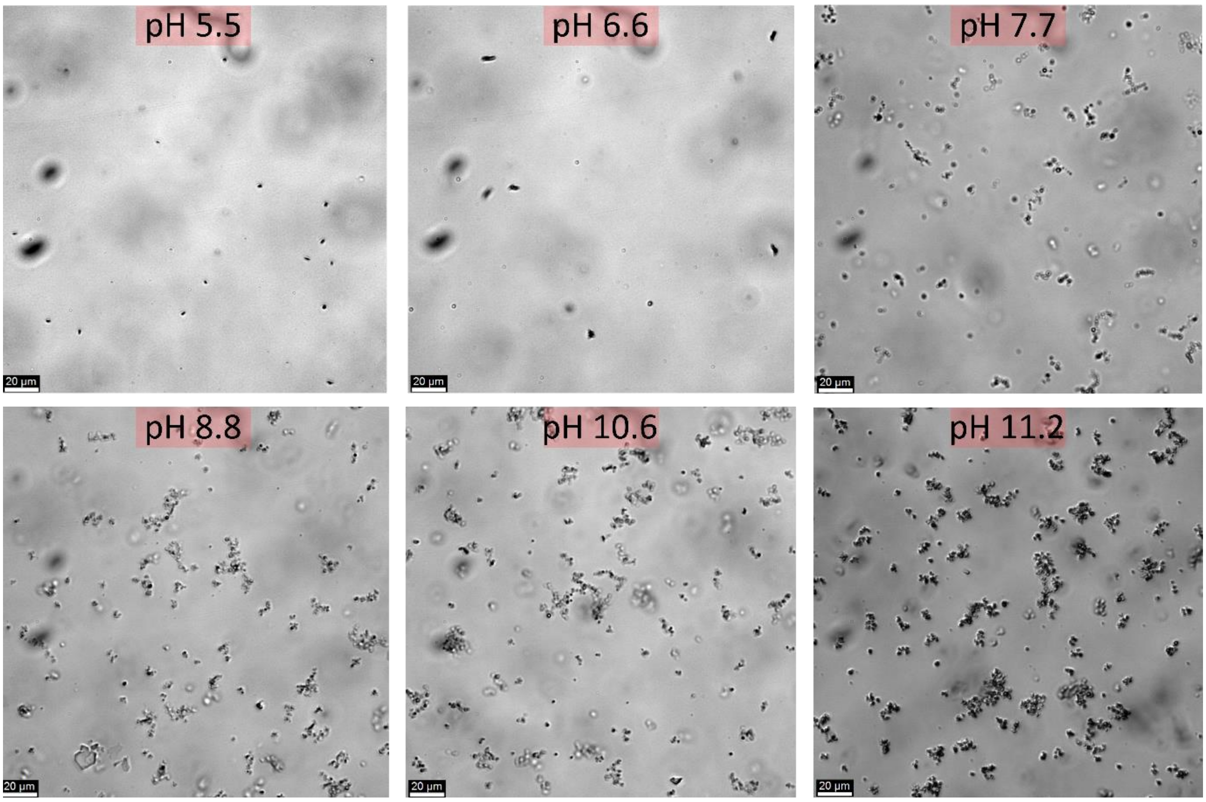

Figure S10. Behavior of FF-NBD peptide (5 mg/mL) in bulk (300 mM NaCl in 5mM HEPES) at different pH. Bright field microscopy images. Scale bar = 20 µm.

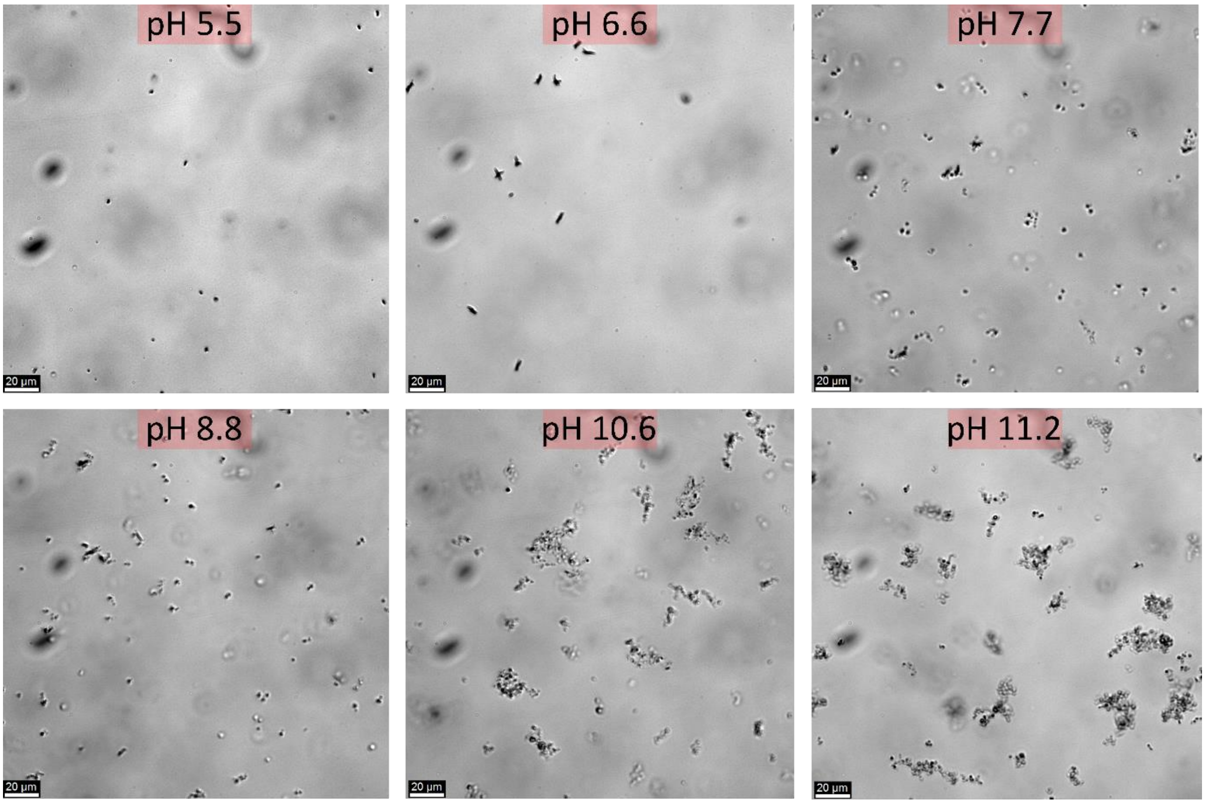

Figure S11. Behavior of FF-NBD peptide (2.5 mg/mL) in bulk (300 mM NaCl in 5mM HEPES) at different pH. Bright field microscopy images. Scale bar = 20 µm.

pH	FF-NBD concentration (mg mL^{-1}) 2.5	5	10
5.5	Low	Low	Low
6.6	Low	Low	Low
7.7	Low	Medium	Medium
8.8	Low	High	High
10.6	Medium	High	High
11.2	High	High	Very high

Figure S12. Qualitative analysis of the tendency of FF-NBD to form aggregates based on the results shown in Figures S9-S11.

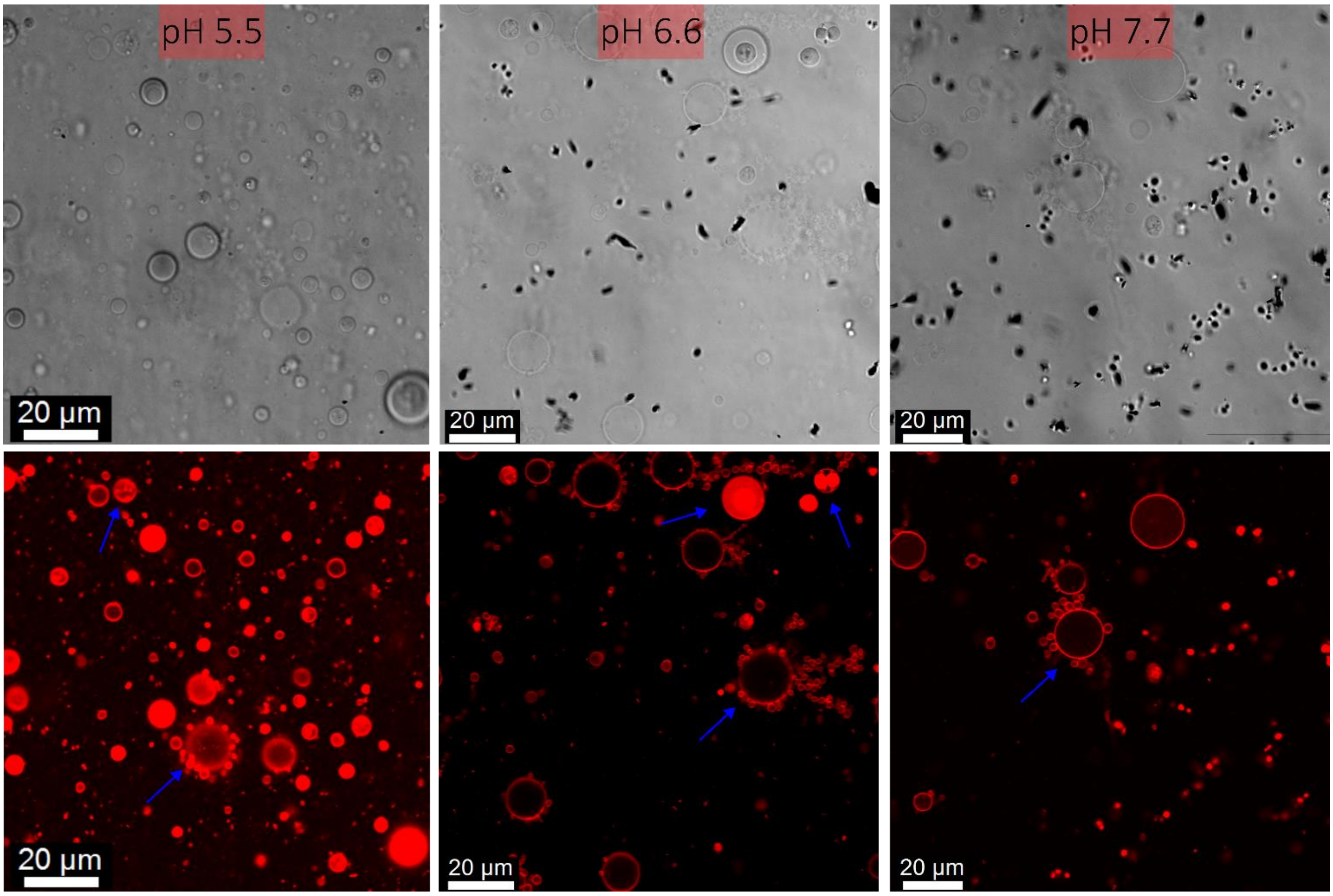

Figure S13. CLSM micrographs of empty polymersomes (without FFM) transferred into chamber containing 10 mg mL^{-1} of FFM peptide. The experiment was repeated at different pHs (5.5, 6.6. and 7.7). Nile Red was used to stain the polymersome membrane. Scale bar = 20 µm.

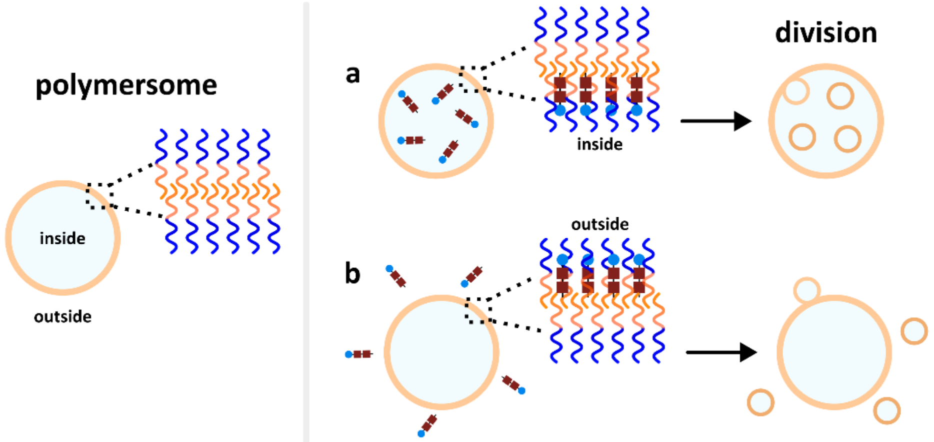

Figure S14. Proposed mechanism of vesicle division. Accumulation of peptides in the vesicle membrane causes asymmetric expansion of either the inner (a) or outer (b) leaflet. This expansion is relieved by bulging and eventual formation of daughter vesicles. Inward and outward bulging and the preferred direction of vesicle division are determined by the localization of the peptide inside and outside the parent vesicles, respectively.

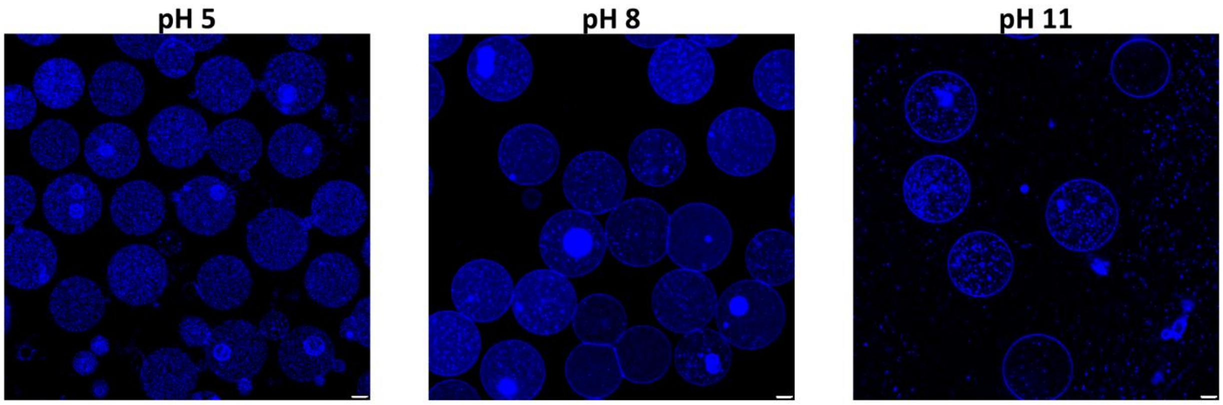

Figure S15. Polymersomes loaded with 10 mg/mL FF-NBD at different pHs. No distinct fiber-like structures were observed outside the vesicles as a result of peptide self-assembly. Scale bar = 10 µm.

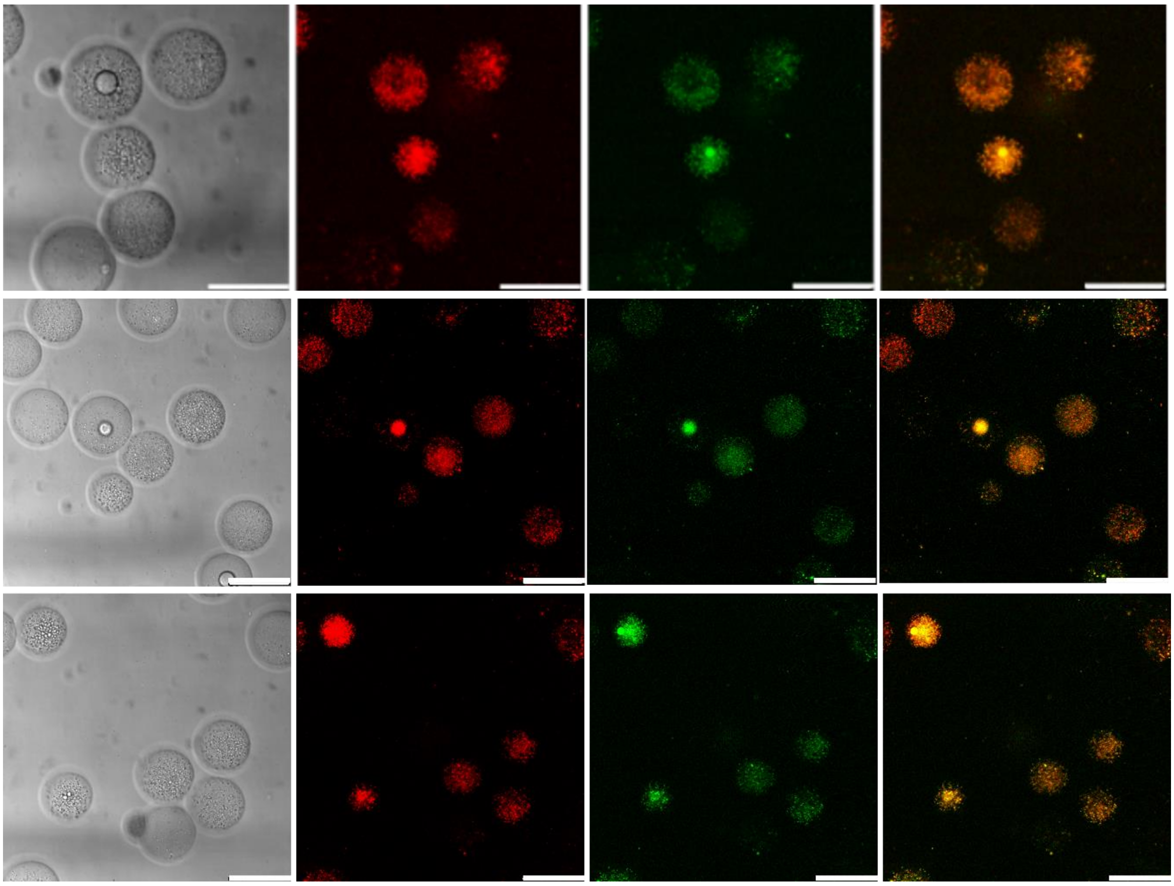

Figure S16. Colocalization of HRP-Cy5 and GOx-FTIC in daughter vesicles. Scale bar = 50 µm.

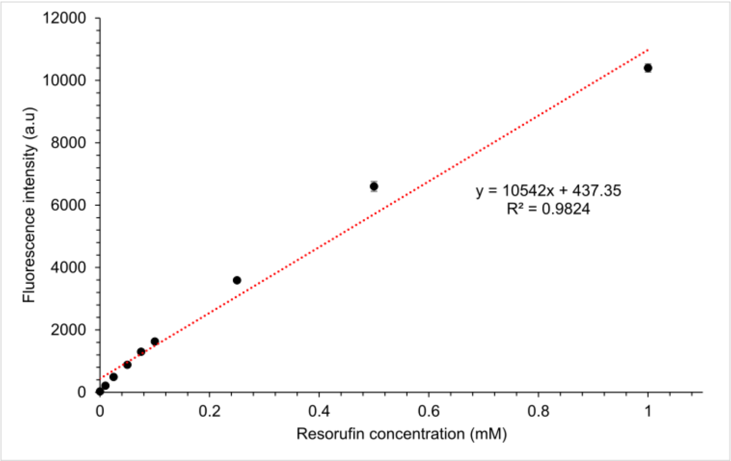

Figure S17. Standard calibration curve of fluorescence intensity vs. [resorufin].

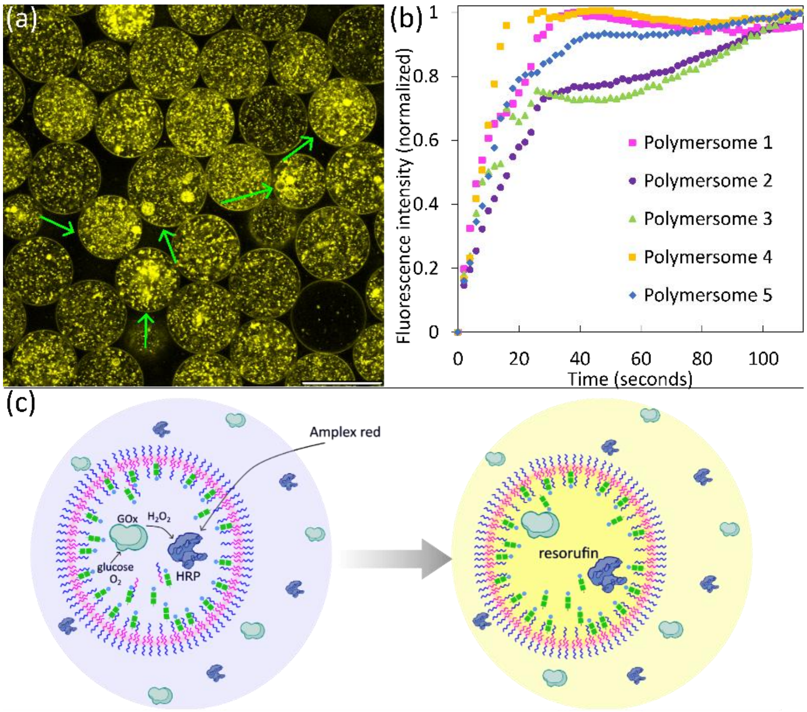

Figure S18. Cascade reaction in a multicompartment polymersome generated by peptide-induced self-division. Top: Confocal micrographs (scale bar = 50 µm) of polymersomes loaded with 10 mg/mL FFM peptide, 0.045 mg/mL GOx, 0.003 mg/mL HRP, and 0.1 M glucose. The reaction was started by the addition of Amplex Red. (b) Kinetics of resorufin production using a confocal microscopy. (c) Schematic of the cascade reaction leading to resorufin production.

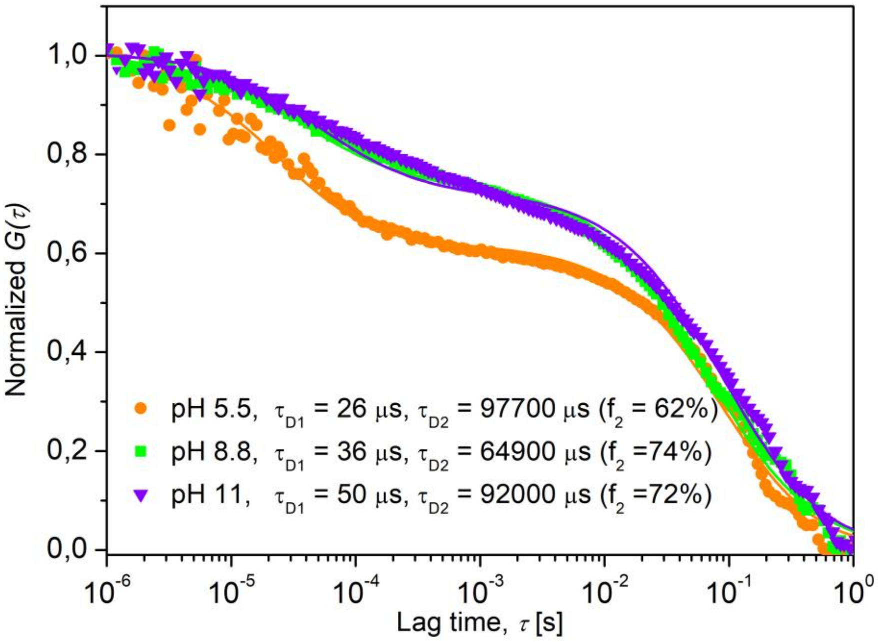

Figure S19. Fluorescence correlation microscopy (FCS) analysis to evaluate FF-NBD diffusion in the lumen (interior) of polymersomes. The solid lines represent fits to eq. S1 using two components (*m* = 2). The presence of large aggregates inside the vesicles complicates data analysis. To estimate the diffusion time of the slow component (aggregates), the diffusion time of the fast component (free peptides) was fixed using the diffusion times of the peptide outside the vesicles (experiments in Figure 3c, top panel). This approach is justified because the diffusion of the small FF-NBD peptide should not change outside or inside the large internal volume of the giant polymersomes. The diffusion time of the slow component (aggregates) t_{D2} originates from large aggregates with diameters around 800 nm.

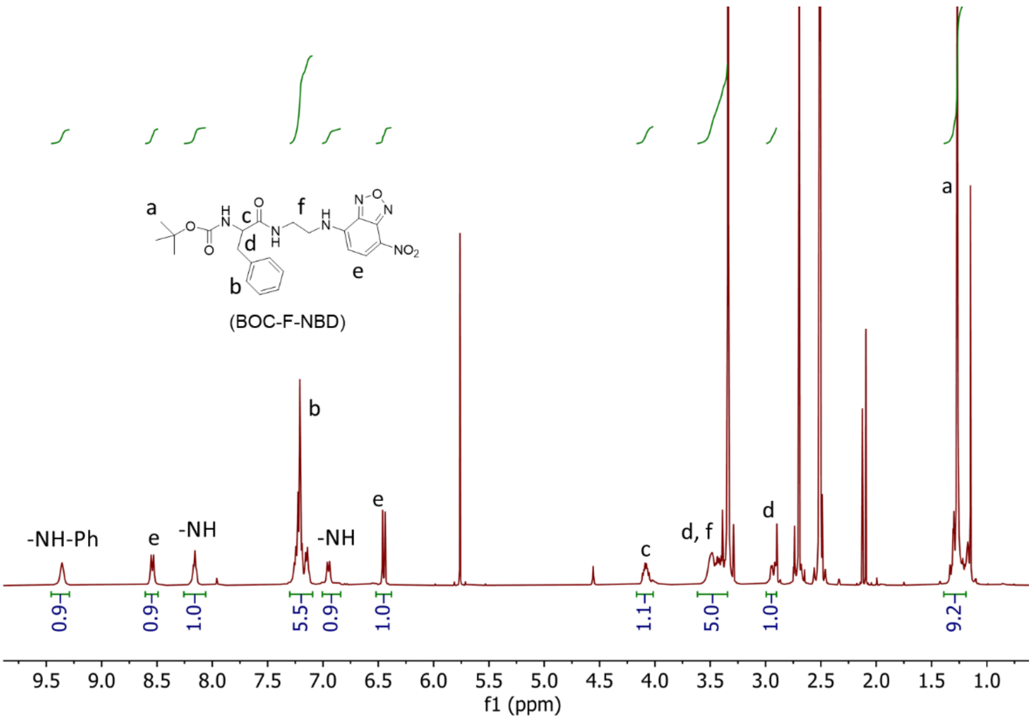

Figure S20. ^{1}H NMR of Boc-F-NBD

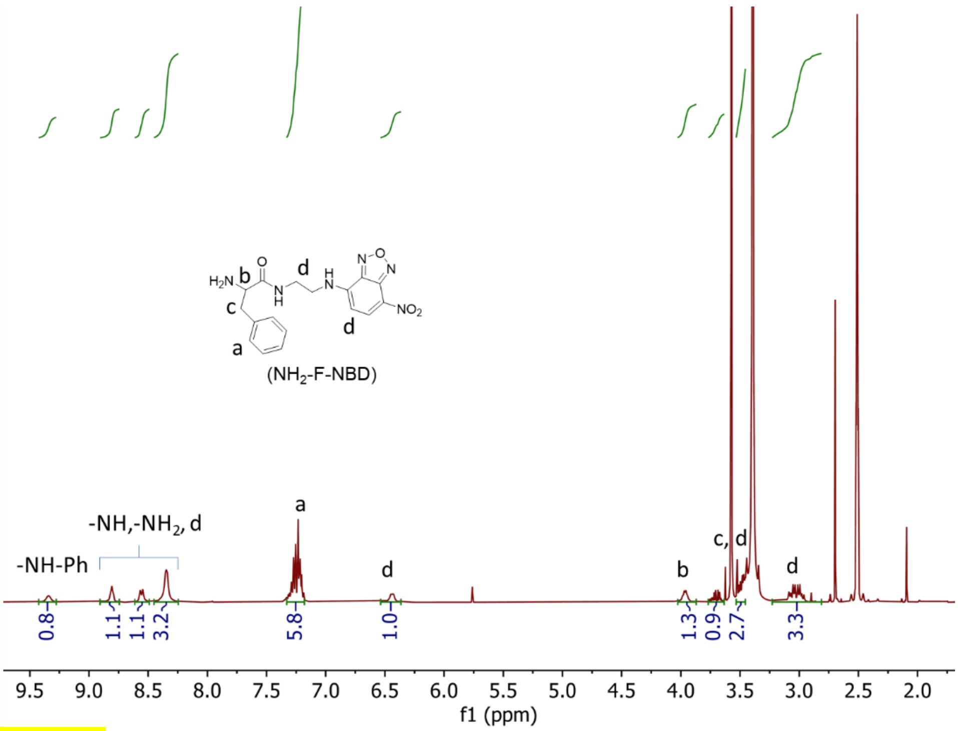

Figure S21. ^{1}H NMR of NH_2-F-NBD

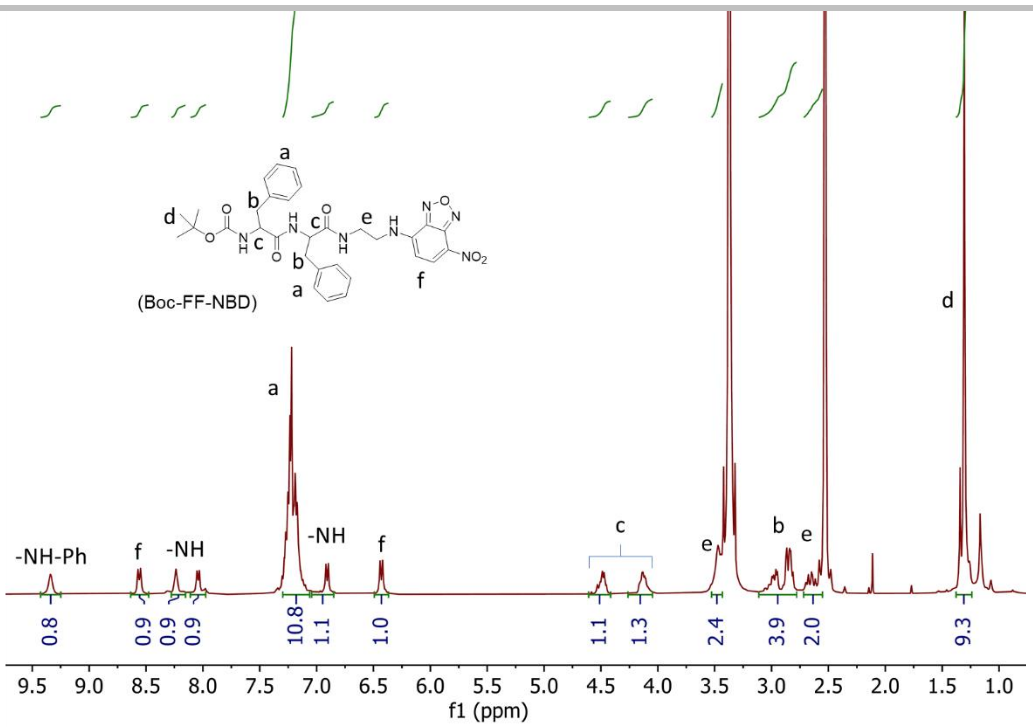

Figure S22. ^{1}H NMR of Boc-FF-NBD

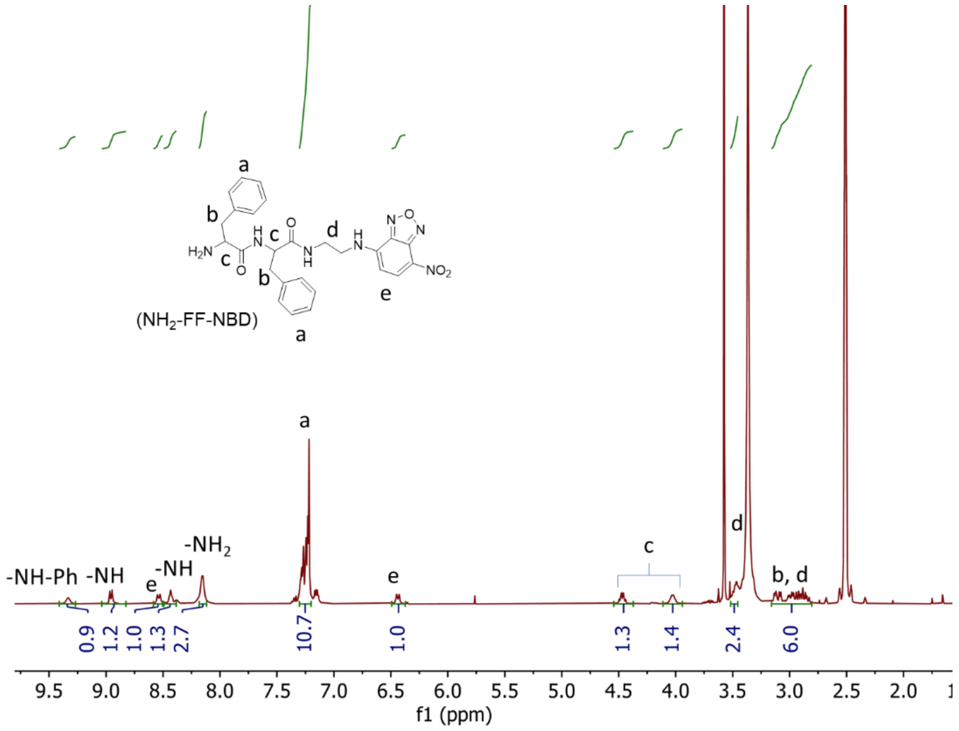

Figure S23. ^{1}H NMR of NH_2-FF-NBD

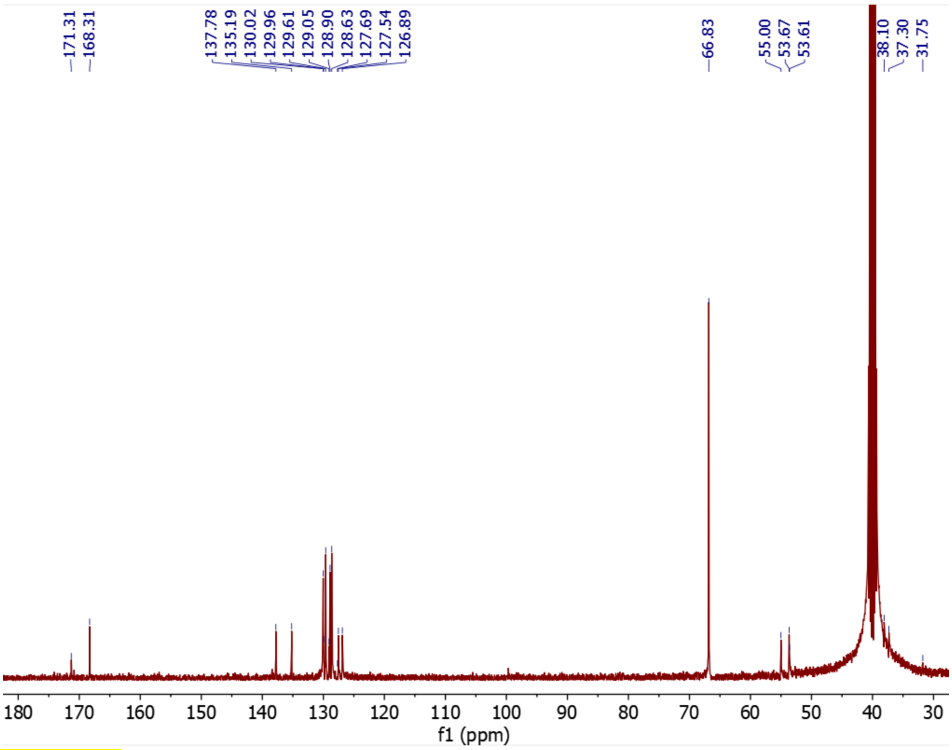

Figure S24. ^{13}C NMR of NH_2-FF-NBD

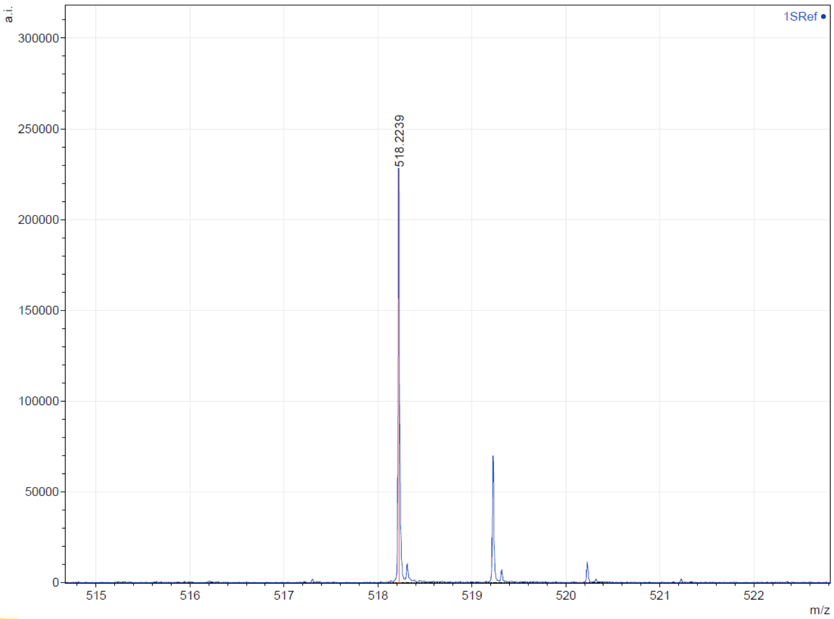

Figure S25. MS of NH_2-FF-NBD

References

[1] S. Cao, T. Ivanov, J. Heuer, C. T. J. Ferguson, K. Landfester, L. Caire da Silva, *Nat Commun* **2024**, *15*.
[2] A. Bandyopadhyay, S. Cambray, J. Gao, *J Am Chem Soc* **2017**, *139*, 871–878.
[3] M. Abbas, W. P. Lipiński, K. K. Nakashima, W. T. S. Huck, E. Spruijt, *Nat Chem* **2021**, *13*, 1046–1054.
[4] R. Rigler, E. S. Elson, *Fluorescence Correlation Spectroscopy - Theory and Applications*, Springer Berlin, Heidelberg, Heidelberg, Germany, **2001**.
[5] A. Vagias, R. Raccis, K. Koynov, U. Jonas, H. J. Butt, G. Fytas, P. Košovan, O. Lenz, C. Holm, *Phys Rev Lett* **2013**, *111*, 088301
